# Method of invigorating spleen and replenishing kidney and resolving phlegm for obesity-type polycystic ovary syndrome: a network meta-analysis and summary of herbal prescription regularity

**DOI:** 10.3389/fmed.2025.1609131

**Published:** 2025-07-28

**Authors:** Yueqiao Lyu, Lian Cai, Mingzhu Li

**Affiliations:** ^1^School of Integrated Chinese and Western Medicine, Anhui University of Chinese Medicine, Hefei, China; ^2^School of Traditional Chinese Medicine, Anhui University of Chinese Medicine, Hefei, China

**Keywords:** invigorating spleen and replenishing kidney and resolving phlegm, obesity-type polycystic ovary syndrome, network meta-analysis, Chinese herbs, prescription regularity

## Abstract

**Objective:**

This study aimed to evaluate the therapeutic efficacy of Invigorating Spleen and Replenishing Kidney and Resolving Phlegm Prescription (ISRKRPP) in treating obesity-type polycystic ovary syndrome (PCOS) and to analyze the prescription regularity through data mining technology.

**Methods:**

Databases were retrieved up to September 30, 2024. RCTs on obesity-type PCOS treated with ISRKRPP and Western medicine (WM) were included. The Risk of Bias 2 tool was used for quality assessment. Network meta-analysis was performed using Stata 14.0. After standardizing herb names, a corresponding herbal database was built in Excel. Based on Excel and R language, the core drugs and prescription rules used in the treatment of obese PCOS were analyzed and visualized.

**Results:**

A total of 14 articles, involving 1,163 patients and 7 prescriptions, were included. Network meta-analysis indicated the following: Qihuang Zengmin Decoction + WM may be optimal for overall efficacy (Confidence in Network Meta-Analysis [CINeMA]: moderate confidence); Cangfu Daotan Decoction + WM showed superiority in reducing BMI (CINeMA: moderate confidence); Method of Regulating and Supplementing Spleen and Kidney + WM most effectively improved HOMA-IR (CINeMA: moderate confidence); Cangfu Daotan Decoction + WM best regulated LH (CINeMA: moderate confidence); Heshi Powder + WM medicine most effectively reduced testosterone (CINeMA: moderate confidence). Herbal Regularity analysis revealed core herbs for the treatment included Rhizoma atractylodis, Poria cocos, Angelica sinensis, *Pinellia ternata*, and Epimedium. The herbal cluster, including Epimedium, Poria cocos, Angelica sinensis, Citrus, Spina gleditsiae, Salviae miltiorrhizae, Rhizoma atractylodis, *Pinellia ternata*, and Rhizoma cyperi may be the core prescription for the treatment of obesity-type PCOS.

**Conclusion:**

Based on the results of the present studies, ISRKRPP combined with WM can improve the effect of obesity-type PCOS. Due to the limitations of the present studies, more clinical studies are needed for further validation.

**Systematic review registration:**

https://www.crd.york.ac.uk/PROSPERO/, identifier CRD42024594257.

## 1 Introduction

PCOS is a common reproductive endocrine illness in reproductive age. It is characterized by chronic anovulation, polycystic ovarian lesions, and hyperandrogenemia (HA), with clinical manifestations including acne, menstrual disorder, infertility, and hirsutism (1). PCOS incidence has risen sharply with a younger onset trend. Globally, the prevalence of PCOS reaches 6–15% among women of reproductive age (2). A Finnish prospective cohort study showed a significant association between body weight and PCOS symptoms in all age groups (3). Currently, the mechanism of obesity-type PCOS remains unclear, but studies have found that for obesity-type PCOS patients, a mere 5% weight reduction can significantly improve ovarian function, adjust reproductive dysfunction, and correct endocrine and metabolic abnormalities (4). Therefore, WM primarily focuses on reducing weight and improving symptoms, rather than a complete cure. Traditional Chinese medicine (TCM) has no specific terminology for PCOS. It is classified as “infertility,” “abdominal masses,” and “amenorrhea” based on its clinical presentations. The method of invigorating the spleen, replenishing the kidney, and resolving phlegm is a treatment method rooted in the theory of TCM and clinical practice. This study aims to comprehensively evaluate the effectiveness of this method in treating obesity-related PCOS through network meta-analysis and to analyze the herbal prescription regularity using data mining techniques, offering an evidence-supported basis for clinical implementation.

## 2 Methods

### 2.1 Registration

The review followed the Preferred Reporting Items for Systematic Reviews and Meta-Analyses (PRISMA) guidelines (5). The study protocol was pre-registered in PROSPERO with No. CRD42024594257.

### 2.2 Inclusion criteria

#### 2.2.1 Study type

The study type included randomized controlled trials (RCTs) with no restriction on language type. Any article that mentions “randomly divided into group” was identified as RCTS.

#### 2.2.2 Study object

According to PCOS guidelines (6, 7), the patients were diagnosed with PCOS, satisfying BMI ≥ 24 (8–12), regardless of disease duration, age, and nationality.

#### 2.2.3 Intervention

The control group received WM without restrictions on the specific type. The experimental group was treated with a prescription for invigorating the spleen, replenishing the kidney, and resolving phlegm based on WM treatment. Prescription formulation included decoction, granules, capsules, and Chinese patent medicine.

#### 2.2.4 Outcome índicator

Main outcome indicators: ① clinical efficacy: clinical efficiency as the evaluation standard, clinical efficiency = number of effective cases/total number of cases × 100% (cure, significant effect, and improvement are in the effective range), ② body mass index (BMI), ③ luteinizing hormone (LH), and ④ testosterone (T).

Secondary outcome indicator: ① adverse events and ② homeostasis model of insulin resistance (HOMA-IR).

### 2.3 Exclusion criteria

① Female subjects have received systemic treatment of reproductive endocrine and metabolic drugs (including but not limited to insulin sensitizers, glucocorticoids, and gonadal modulators) within the past 3 months or are pregnant or postpartum lactating; ② prescription no effect for invigorating spleen, replenishing kidney, and resolving phlegm; ③ duplicate publications and other suspected duplicate literature; ④ the study subjects included non-PCOS patients and untreated PCOS patients; ⑤ literature with incomplete full-text data and could not be obtained by contacting the authors; ⑥ reviews, animal studies, conferences, patents, etc.

### 2.4 Literature search strategy

Databases searched included PubMed, Embase, the Cochrane Library, Web of Science, China Knowledge Network Infrastructure (CNKI), Wanfang Data, China Science and Technology Journal Database (VIP), and China Biomedical Literature Database (CBM). Literature searches were conducted by combining MeSH terms and free words, with the date from the earliest available date to September 30, 2024. Taking PubMed for instance, the detailed search strategy is presented as follows:

#1 “Obesity” [MeSH Terms]

#2 “Polycystic Ovary Syndrome” [MeSH Terms]

#3 “polycystic ovarian syndrome” [Title/Abstract] OR “polycystic ovary syndrome 1” [Title/Abstract] OR “sclerocystic ovarian degeneration” [Title/Abstract] OR “sclerocystic ovaries” [Title/Abstract] OR “sclerocystic ovary syndrome” [Title/Abstract] OR “stein leventhal syndrome” [Title/Abstract]

#4 #2 OR #3

#5 “medicine, chinese traditional” [MeSH Terms]

#6 “Chinese Medicine, Traditional” [Title/Abstract] OR “Chinese Traditional Medicine” [Title/Abstract] OR “Chung I Hsueh” [Title/Abstract] OR “Traditional Chinese Medicine” [Title/Abstract] OR “Traditional Medicine, Chinese” [Title/Abstract] OR “Traditional Tongue Assessment” [Title/Abstract] OR “Traditional Tongue Diagnosis” [Title/Abstract] OR “Zhong Yi Xue” [Title/Abstract] OR “TCM” [Title/Abstract]

#7 “drugs, chinese herbal” [MeSH Terms]

#8 “Chinese Drugs, Plant” [Title/Abstract] OR “Chinese Herbal Drugs” [Title/Abstract] OR “Plant Extracts, Chinese” [Title/Abstract] OR “Chinese Herbal” [Title/Abstract] OR “Tang” [Title/Abstract] OR “Fang” [Title/Abstract] OR “Prescription” [Title/Abstract]

#9 #5 OR #6 OR #7 OR #8

#10 “Randomized Controlled Trial” [Publication Type]

#11 “Randomized Controlled Trials as Topic” [MeSH Terms] OR “RCT” [Title/Abstract] OR “clinical trial” [Title/Abstract] OR “Randomly” [Title/Abstract] OR “controlled clinical trial” [Title/Abstract] OR “Randomized” [Title/Abstract] OR “clinical study” [Title/Abstract]

#12 #10 OR #11

#13 #1 AND #4 AND #9 AND #12

The specific retrieval strategies for the other databases can be found in the Supplementary materials.

### 2.5 Data extraction

All retrieved literature was imported into EndNote, where duplicates were identified and removed. Two researchers independently screened the literature according to the inclusion and exclusion criteria. In the initial screening, titles and abstracts were reviewed to exclude irrelevant studies. In the re-screening, full texts were examined to finalize the included literature. Any disagreements were resolved by a third researcher. Detailed data were extracted from the final included literature, including study title, first author, trial protocol, publication year, sample size, case attrition, baseline characteristics, prescription composition, and outcome indicators. Extracted data were cross-checked by both researchers, and disagreements were resolved by a third party.

### 2.6 Assessment for risk of bias

Risk of bias was assessed using the Cochrane Collaboration Risk of Bias Tool version 2.0, categorizing studies as “Low risk,” “High risk,” or “Some concerns.” The evaluation covered randomization, deviations from the planned intervention, missing data, outcome measurement, and selective reporting. Two independent investigators reviewed the studies and cross-checked their findings, and a third investigator resolved any disagreements.

### 2.7 Statistical analysis

Network meta-analysis was based on the frequency-based framework and used Stata 14.0 software for data analysis. The dichotomous outcome indicators were described by relative risk (RR), and continuous variables were described by mean difference (MD), while 95% probability density confidence intervals (95% CIs) were applied to describe the confidence intervals of each effect size. Cochrane’s *Q* test combined with *Ι^2^* was used to evaluate the included studies’ heterogeneity. If *p* ≥ 0.05 and *Ι^2^* < 50%, the heterogeneity between studies was low, and a fixed-effects model was applied to combine the effect sizes; if *p* < 0.05 and *Ι^2^* ≥ 50%, high heterogeneity was assumed, and a random-effects model was used. For the effect indicators with significant heterogeneity, further sensitivity or subgroup analyses were conducted to identify relevant literature and minimize heterogeneity and then implement network meta-analysis. The network evidence map was generated using the “network” command in Stata. When the evidence network formed a closed loop, the inconsistency test was performed. Among the outcome indicators, the surface under the cumulative ranking curve (SUCRA) was used to rank the interventions according to the magnitude of the values. Stata 14.0 software was used to draw the publication bias funnel plot.

### 2.8 Rating the confidence of evidence

This study confidence assessment adopted an online software—CINeMA (13) (Confidence In Network Meta-Analysis). CINeMA online applications can be accessed directly through their official website: https://cinema.ispm.unibe.ch/. It explores the confidence level of the network results by evaluating six domains: within-study bias, report bias, indirectness, imprecision, heterogeneity, and incoherence. Except for the publication bias, which was reported as “suspected” or “undetected,” the rest were rated as “no concerns,” “some concerns,” or “major concerns.” The confidence of evidence by CINeMA was rated as high, moderate, low, or very low.

## 3 Results

### 3.1 Study selection

**Figure 1:**
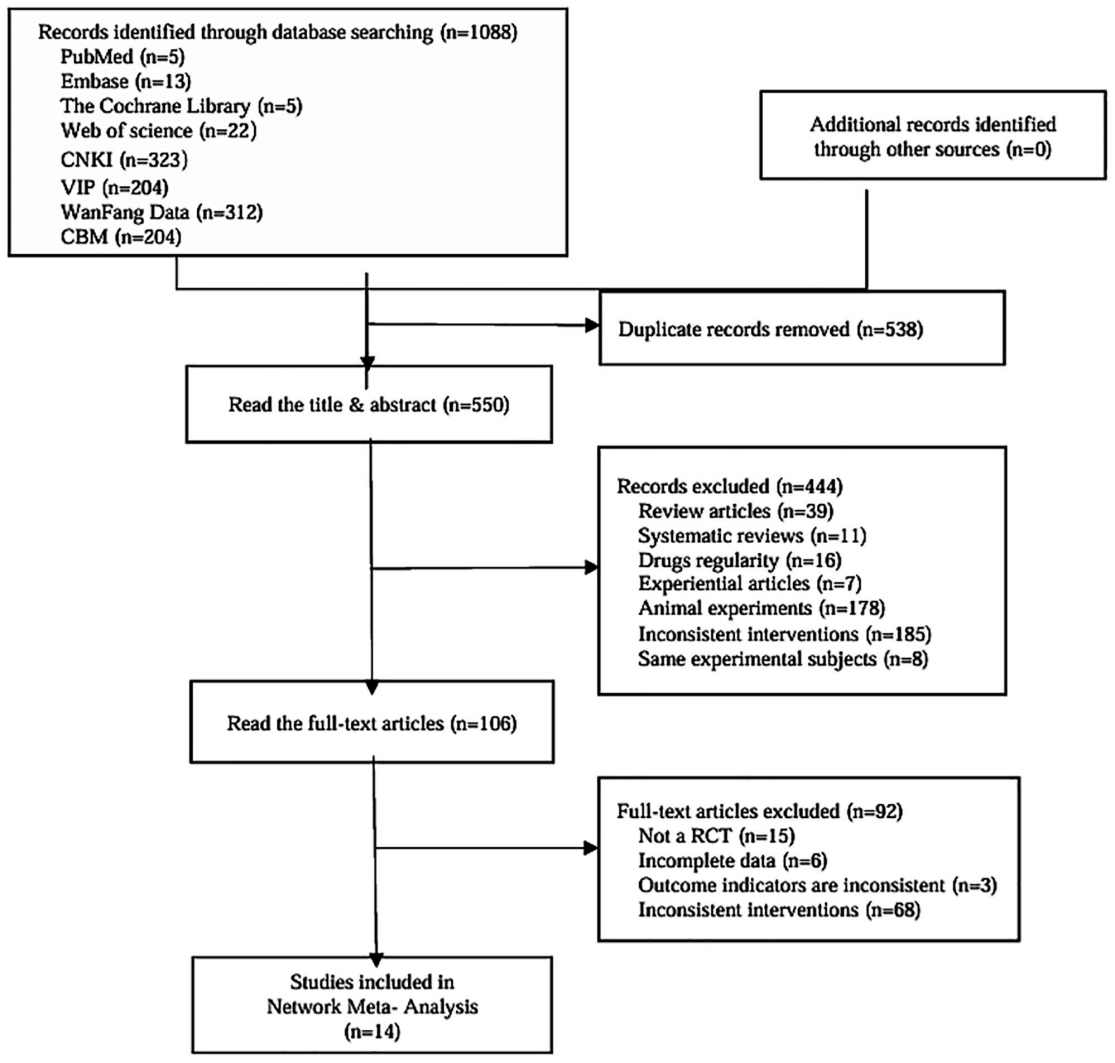
Article screening process.

The preliminary systematic search obtained 1,088 relevant articles, and 550 articles were retained after removing duplicates. Further screening by full-text review excluded articles that did not meet the inclusion criteria or lacked complete research data, and 14 articles (14–27) were finally included in the study. The PRISMA flow chart presents the study selection as Figure 1, and the search formulas of each database are shown in the additional materials.

### 3.2 Study characteristics

A total of 14 RCTs involving 1,163 participants were included, with 581 cases in the control group and 582 cases in the experimental group. The literature was all in Chinese, published from 2015 to 2024, involving 7 prescriptions for invigorating spleen, replenishing kidney, and resolving phlegm, including 3 for Prescription of Invigorating Kidney and Dissipating Phlegm (PIKDP), 4 for Cangfu Daotan Decoction (CFDTD), 3 for Method of Regulating and Supplementing Spleen and Kidney (MRSSK), 1 for Promoting Ovulation Decoction (POD), 1 for Qihuang Zengmin Decoction (QHZMD), 1 for Self-made Tonifying Qi and Removing Turbidity Decoction (STQRTD), and 1 for Heshi Powder (HSP). The baseline data of the two groups were comparable in all the literature. The control group received WM, and the experimental group received an oral Chinese prescription based on WM. All the study treatment courses were three menstrual cycles or more and are detailed in Table 1.

**Table 1:** Basic features of the included literature.

Literature resources	Sample size		Age		Disease duration		Intervention		Treatment duration	Outcome indicator	Adverse reaction	
	C	T	C	T	C	T	C	T			C	T
Liu et al. (14)	20	20	25.65 ± 4.40	26.35 ± 4.88	—	—	WM	PIKDP+WM	3	①②③④⑤⑥	Severe vomiting and diarrhea in two patients on metformin	No adverse reactions
Lu et al. (15)	55	55	30.1 ± 4.0	30.0 ± 3.9	5.2 ± 1.9	5.2 ± 1.9	WM	PIKDP+WM	3	③④	No reported	No reported
Hou et al. (16)	32	32	28.21 ± 3.57	26.97 ± 2.07	2.99 ± 1.34	2.95 ± 1.34	WM	PIKDP+WM	3	①②③④⑤⑥	No adverse reactions	No adverse reactions
Guo et al. (17)	40	40	27.9 ± 1.3	29.8 ± 2.1	5.9 ± 2.1	3.6 ± 1.2	WM	CFDTD+WM	3	①⑤⑥	No reported	No reported
Huang et al. (18)	42	42	24.59 ± 4.92	24.87 ± 4.67	—	—	WM	CFDTD+WM	3	①②③	Nausea and vomiting in two patients, dyspepsia in one patient	Nausea and vomiting in 2 patients, intermittent diarrhea in 2 patients
Shan et al. (19)	60	60	28.41 ± 2.21	28.23 ± 2.18	5.21 ± 1.24	5.19 ± 1.19	WM	CFDTD+WM	3	①③	No reported	No reported
Zeng et al. (20)	36	36	26.12 ± 4.97	27.54 ± 5.32	3.96 ± 0.99	4.08 ± 1.14	WM	POD+WM	3	①	No reported	No reported
Zhang et al. (21)	40	40	32.33 ± 3.17	31.89 ± 3.24	4.83 ± 0.66	4.76 ± 0.65	WM	MRSSK+WM	6	①③⑤⑥	No reported	No reported
Li et al. 2020 (22)	35	35	28.64 ± 3.23	28.76 ± 3.25	2.07 ± 0.14	2.11 ± 0.16	WM	MRSSK+WM	3	①②④⑤⑥	No adverse reactions	No adverse reactions
Liu et al. (23)	49	49	28.29 ± 6.13	28.63 ± 6.76	3.13 ± 0.95	3.82 ± 0.86	WM	MRSSK+WM	3	④⑤⑥	No reported	No reported
Liang et al. (24)	60	60	30.14 ± 2.01	30.24 ± 2.43	4.51 ± 2.24	4.56 ± 2.13	WM	CFDTD+WM	3	①⑤⑥	No reported	No reported
Zhou et al. (25)	30	30	27.80 ± 4.35	27.20 ± 3.73	4.1 ± 1.01	4.5 ± 1.26	WM	HSP + WM	3	①②③④⑤⑥	No adverse reactions	Slight nausea and bloating in 2 patients on medication, relieved by symptomatic treatment
Bai et al. (26)	37	38	25.57 ± 5.44	26.45 ± 5.16	—	—	WM	QHZMD+WM	3	②④⑤⑥	Nausea and vomiting occurred in two cases, without special treatment, and improved after 30 min of rest.	Nausea and vomiting occurred in 3 cases, without special treatment, and improved after 30 min of rest.
Wang et al. (27)	45	45	—	—	—	—	WM	STQRTD+WM	3	①③	No reported	No reported

T, treatment group; C, control group; Treatment duration, menstrual cycle; WM, western medicine; CFDTD, Cangfu Daotan Decoction; HSP, Heshi Powder; STQRT, Self-made Tonifying qi and Removing turbidity decoction; POD, Promoting Ovulation Decoction; QHZMD, Qihuang Zengmin Decoction; PIKDP—Prescription of Invigorating Kidney and Dissipating Phlegm; MRSSK, Method of Regulating and Supplementing Spleen and Kidney; ① clinical efficacy; ② adverse reaction; ③ BMI; ④ HOMA-IR; ⑤ LH; ⑥ T.

### 3.3 Risk of bias

**Figure 2:**
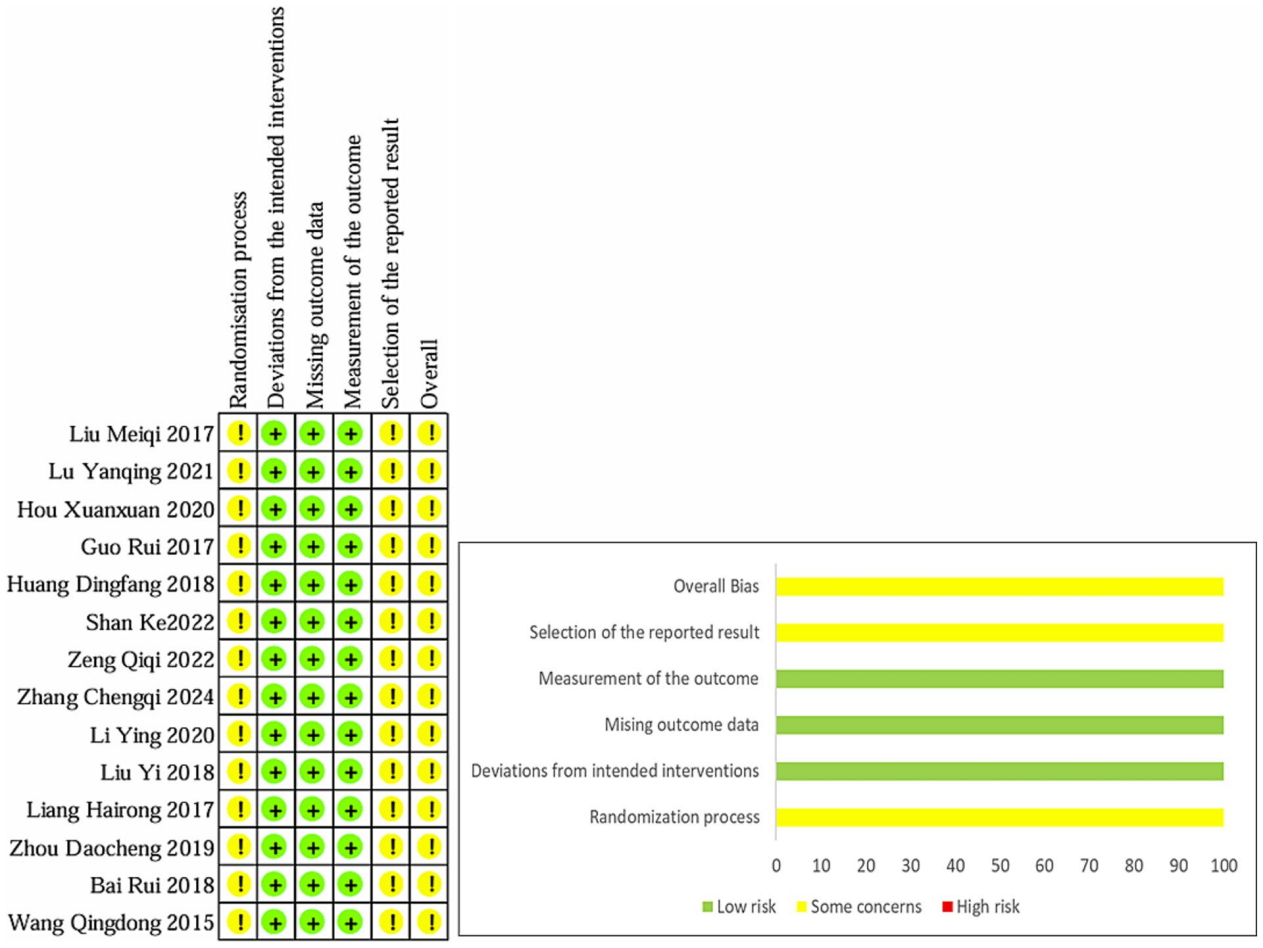
Percentages of items of included articles that produced risks of bias.

ROB2.0 was used to evaluate the quality of the included literature. All 14 RCTs mentioned random allocation, seven of them (18–20, 22, 23, 25, 26) used randomized numeric tables, one (21) used the order of hospital treatment, and one (24) used the method of treatment. None of the 14 RCTs mentioned blinding, so the bias in the randomization process was rated as “Some concerns.” All literature intervention allocation was rated as “low risk.” All literature was rated as “low risk” with complete outcome data, and all outcome measures were appropriately rated as “low risk.” Due to insufficient evidence to determine the existence of selective reporting of results, all literature was rated as “Some concerns” regarding the potential of selective reporting bias and is detailed in Figure 2.

### 3.4 Evidence network

**Figure 3:**
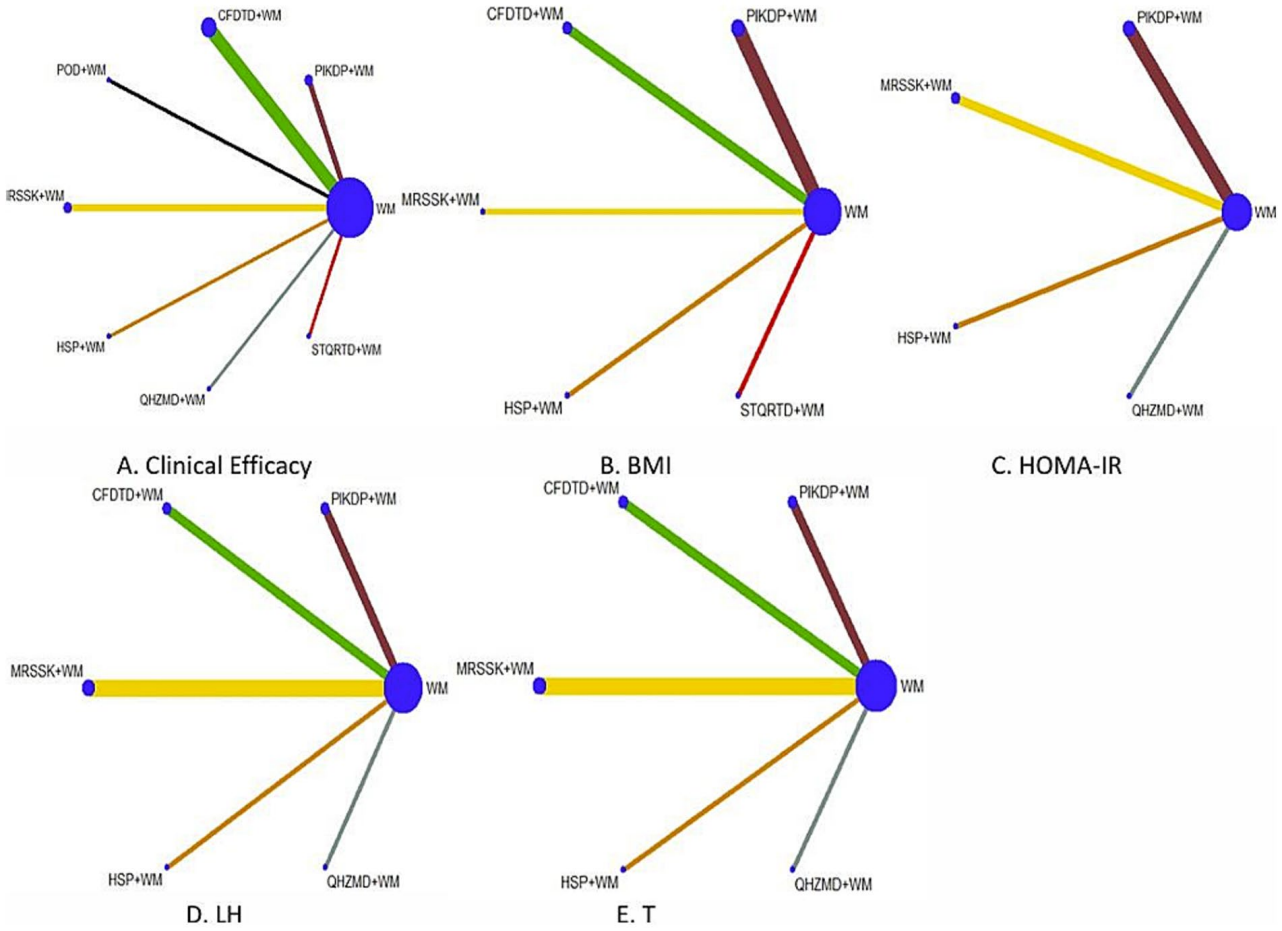
Evidence network of each outcome indicator.

The evidence network is shown in Figure 3, in which the blue dots represent different interventions, the size of the dots represents the sample size of the intervention measures, and the diameter of the dots is positively correlated with the sample size. The thickness of the line between two dots represents the number of studies included for each intervention, the thickness positively correlating with the number of studies. Evidence network diagrams for each outcome indicator show that there are no closed loops and no direct comparisons between all interventions, which aligns with the consistency model, not require an inconsistency test.

### 3.5 Clinical efficacy

#### 3.5.1 Meta-analysis

Twelve (14, 16–22, 24–26) RCTs reported clinical efficacy involving seven prescriptions. Meta-analysis showed significant heterogeneity among the studies (*Ι^2^* = 44%, *p* = 0.050), so the random effect model was used to merge the effect size. The analysis results showed that the treatment of ISRKRPP+WM was better than simple WM in improving the clinical efficacy of obese PCOS, and the difference showed statistical significance (RR = 1.23, 95% CI [1.15, 1.31], *p* < 0.05). Subgroup analysis was performed according to BMI before treatment, treatment course, specific Chinese medicine prescription category of the experimental group, and type of WM used, and no significant change in heterogeneity was found.

#### 3.5.2 Network meta-analysis

The results demonstrated that PIKDP+WM ( logeRR =1.50, 95% CI [0.49, 2.50]), CFDTD+WM ( logeRR =1.45, 95% CI [0.86, 2.05]), MRSSK+WM ( logeRR =1.29, 95% CI [0.30, 2.28]), and QHZMD+WM ( logeRR =2.16, 95% CI [0.57, 3.74]) in the treatment of obese PCOS were better than simple WM treatment in improving the total clinical efficacy rate, and the difference was statistically significant. There was no significant statistical difference between the two interventions (see Table 2).

**Table 2:** Network meta-analysis of clinical efficacy.

Intervention	$\log_{e}\mathrm{RR}\;$ (95% CI)							
	PIKDP+WM	CFDTD+WM	POD+WM	MRSSK+WM	HSP + WM	QHZMD+WM	STQRTD+WM	WM
PIKDP+WM	0							
CFDTD+WM	0.04 (−1.13, 1.21)	0						
POD+WM	0.24 (−1.37, 1.85)	0.20 (−1.20, 1.59)	0					
MRSSK+WM	0.21 (−1.20, 1.62)	0.17 (−0.99, 1.32)	−0.03 (−1.63, 1.57)	0				
HSP + WM	0.43 (−1.14, 2.01)	0.39 (−0.96, 1.74)	0.20 (−1.55, 1.94)	0.22 (−1.34, 1.79)	0			
QHZMD+WM	−0.66 (−2.54, 1.21)	−0.70 (−2.39, 0.99)	−0.90 (−2.92, 1.12)	−0.87 (−2.74, 1.00)	−1.09 (−3.09, 0.90)	0		
STQRTD+WM	−0.52 (−3.68, 2.64)	−0.56 (−3.61, 2.49)	−0.76 (−4.00, 2.49)	−0.73 (−3.88, 2.42)	−0.95 (−4.18, 2.28)	0.14 (−3.24, 3.53)	0	
WM	1.50 (0.49, 2.50)	1.45 (0.86, 2.05)	1.26 (0.00, 2.52)	1.29 (0.30, 2.28)	1.06 (−0.15, 2.28)	2.16 (0.57, 3.74)	2.01 (−0.98, 5.01)	0

#### 3.5.3 Rank of SUCRA

**Figure 4:**
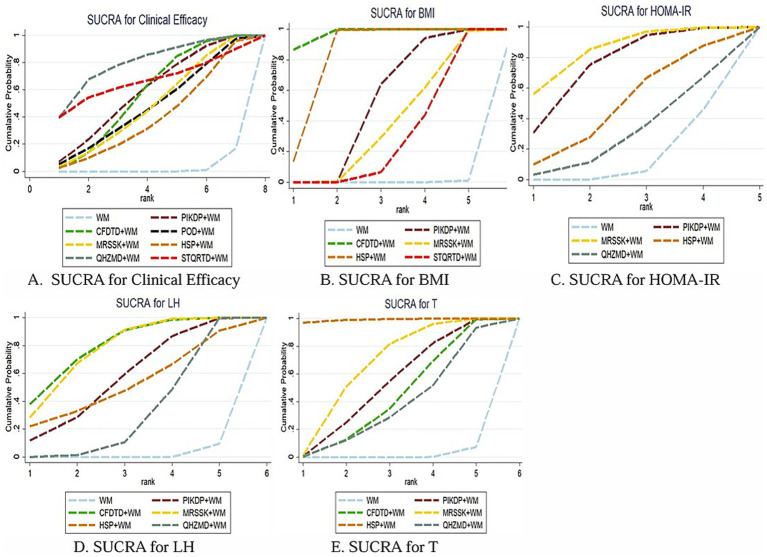
SUCRA ranking for each outcome index.

The order of SUCRA of the seven prescriptions to enhance the clinical efficiency effect was QHZMD+WM (SUCRA = 80.2%) > STQRTD+WM (SUCRA = 66.5%) > PIKDP+WM (SUCRA = 58.1%) > CFDTD+WM (SUCRA = 56.8%) > MRSSK+WM (SUCRA = 48.3%) > POD+WM (SUCRA = 47.6%) > HSP + WM (SUCRA = 39.9%) > WM (SUCRA = 2.6%) (see Figure 4).

### 3.6 BMI

#### 3.6.1 Meta-analysis

Eight (14–16, 18, 19, 21, 25, 26) RCTs reported BMI, involving five prescriptions. Meta-analysis showed that there was significant heterogeneity among the studies (*Ι^2^* = 82.8%, *p* < 0.001). The random effect model was used to combine the effect size. The analysis results showed that the treatment of ISRKRPP+WM was better than simple WM in reducing BMI in obese PCOS patients. The difference showed statistically significant (MD = −1.02, 95% CI [−1.19, −0.85], *p* < 0.05). Subgroup analysis was performed according to BMI before treatment, treatment course, specific Chinese medicine prescription category of the experimental group, and type of WM used and did not find any significant change in heterogeneity. The literature was excluded one by one, and the heterogeneity was reduced by the exclusion of the literature (19) (*Ι^2^* = 52.8%, *p* = 0.048).

#### 3.6.2 Network meta-analysis

The results showed that PIKDP+WM (MD = −1.51, 95% CI [−2.06, −0.96]), CFDTD+WM (MD = −4.53, 95% CI [−6.18, −2.88]), MRSSK+WM (MD = −1.21, 95% CI [−2.23, −0.19]), HSP + WM (MD = −3.42, 95% CI [−4.61, −2.23]), and STQRTD+WM (MD = −1.05, 95% CI [−1.54, −0.56]) were statistically significant differences in reducing BMI, which were superior to WM alone in the treatment of obesity-type PCOS. Pairwise comparisons of the interventions revealed that CFDTD+WM (MD = -3.02, 95% CI [−4.75, −1.28]) and HSP + WM (MD = −1.91, 95% CI [−3.21, −0.60]) were superior to the treatment of PIKDP+WM, with a statistically significant difference; HSP + WM (MD = −2.21, 95% CI [−0.56]) was superior to the treatment of MRSSK+WM, with a statistically significant difference. There was no statistically significant difference in pairwise comparison among the other interventions (see Table 3).

**Table 3:** Network meta-analysis of BMI.

Intervention	MD (95% CI)					
	PIKDP+WM	CFDTD+WM	MRSSK+WM	HSP + WM	STQRTD+WM	WM
PIKDP+WM	0					
CFDTD+WM	3.02 (1.28, 4.75)	0				
MRSSK+WM	−0.30 (−1.47, 0.86)	−3.32 (−5.26, −1.38)	0			
HSP + WM	1.91 (0.60, 3.21)	−1.11 (−3.14, 0.92)	2.21 (0.64, 3.78)	0		
STQRTD+WM	−0.46 (−1.20, 0.28)	−3.48 (−5.20, −1.76)	−0.16 (−1.30, 0.98)	−2.37 (−3.65, −1.09)	0	
WM	−1.51 (−2.06, −0.96)	−4.53 (−6.18, −2.88)	−1.21 (−2.23, −0.19)	−3.42 (−4.61, −2.23)	−1.05 (−1.54, −0.56)	0

#### 3.6.3 Rank of SUCRA

The order of SUCRA for the five prescriptions in reducing BMI was CFDTD+WM (SUCRA = 97.1%) > HSP + WM (SUCRA = 82.7%) > PIKDP+WM (SUCRA = 51.7%) > MRSSK+WM (SUCRA = 38.3%) > STQRTD+WM (SUCRA = 29.9%) > WM (SUCRA = 0.2%) (see Figure 4).

### 3.7 HOMA-IR

#### 3.7.1 Meta-analysis

Seven (14–16, 22, 23, 25, 26) RCTs reported HOMA-IR involving four Chinese medicine prescriptions. The traditional meta-analysis showed that the heterogeneity among the studies was low (*Ι^2^* = 9.3%, *p* = 0.358), so the fixed effect model was used to pool the effect size. The analysis results showed that the treatment of ISRKRPP combined with WM was better than simple WM in reducing HOMA-IR in obese PCOS patients. The difference was statistically significant (MD = −0.69, 95% CI [−0.78, −0.43], *p* < 0.05).

#### 3.7.2 Network meta-analysis

The results showed that PIKDP (MD = −0.73, 95% CI [0.15, 1.32]), MRSSK (MD = −0.88, 95% CI [−1.55, −0.22]) + WM were superior to simple WM in improving HOMA-IR in obese PCOS patients, and the difference was statistically significant in all of them. Pairwise comparisons between the interventions showed no statistically significant differences (see Table 4).

**Table 4:** Network meta-analysis of HOMA-IR.

Intervention	MD (95% CI)				
	PIKDP+WM	MRSSK+WM	HSP + WM	QHZMD+WM	WM
PIKDP+WM	0				
MRSSK+WM	0.15 (−0.67, 0.97)	0			
HSP + WM	−0.33 (−1.29, 0.62)	−0.48 (−1.49, 0.52)	0		
QHZMD+WM	−0.58 (−1.55, 0.38)	−0.73 (−1.76, 0.29)	−0.25 (−1.33, 0.83)	0	
WM	−0.73 (−1.32, −0.15)	−0.88 (−1.55, −0.22)	−0.40 (−1.15, 0.35)	−0.15 (−0.93, 0.63)	0

#### 3.7.3 Rank of SUCRA

The order of SUCRA for the four prescriptions in reducing HOMA-IR was MRSSK+WM (SUCRA = 84.2%)>PIKDP+WM (SUCRA = 75.1%) > HSP + WM (SUCRA = 49.1%) > QHZMD+WM (SUCRA = 28.9%)> > WM (SUCRA = 12.7%) (see Figure 4).

### 3.8 LH

#### 3.8.1 Meta-analysis

Nine (14, 16, 17, 21–26) RCTs reported LH, including five Chinese medicine prescriptions. The traditional meta-analysis showed that there was significant heterogeneity among the studies (*Ι^2^* = 90.3%, *p* < 0.001). The random effect model was used to combine the effect size. The analysis results showed that the treatment of ISRKRPP+WM was better than simple WM in improving LH in obese PCOS patients, and the difference proved to be statistically significant (MD = −0.61, 95% CI [−0.76, −0.45], *p* < 0.05). Subgroup analysis was performed according to BMI before treatment, treatment course, specific Chinese medicine prescription category of the experimental group, and type of WM used and did not find any significant change in heterogeneity. Excluding the literature one by one, it was found that the heterogeneity decreased after excluding (17) *Ι^2^* = 71.2%, *p* < 0.001; after excluding the literature (22) *Ι^2^* = 87.6%, *p* < 0.001; and after excluding (17, 22), the heterogeneity decreased significantly (*Ι^2^* = 0.0%, *p* = 0.46).

#### 3.8.2 Network meta-analysis

The results showed that PIKDP+WM (MD = −1.98, 95% CI [−3.29, −0.66]), CFDTD+WM (MD = −2.60, 95% CI [−3.68, −1.52]), MRSSK+WM (MD = -2.51, 95% CI [−3.35, −1.66]), and QHZMD+WM (MD = −1.28, 95% CI [−2.11, −0.45]) were statistically significant differences in reducing LH, which were superior to the WM alone in treating obese PCOS. Pairwise comparisons of the interventions revealed that MRSSK+WM (MD = −1.23, 95% CI [−2.42, −0.04]) was superior to the therapeutic efficacy of QHZMD+WM, and the difference was statistically significant. There was no statistically significant difference in pairwise comparison among the other interventions (see Table 5).

**Table 5:** Network meta-analysis of LH.

Intervention	MD (95% CI)					
	PIKDP+WM	CFDTD+WM	MRSSK+WM	HSP + WM	QHZMD+WM	WM
PIKDP+WM	0					
CFDTD+WM	0.62 (−1.08, 2.33)	0				
MRSSK+WM	0.53 (−1.03, 2.10)	−0.09 (−1.46, 1.28)	0			
HSP + WM	−0.20 (−3.19, 2.80)	−0.82 (−3.72, 2.08)	−0.73 (−3.55, 2.09)	0		
QHZMD+WM	−0.70 (−2.26, 0.86)	−1.32 (−2.68, 0.04)	−1.23 (−2.42, −0.04)	−0.50 (−3.32, 2.32)	0	
WM	−1.98 (−3.29, −0.66)	−2.60 (−3.68, −1.52)	−2.51 (−3.35, −1.66)	−1.78 (−4.47, 0.91)	−1.28 (−2.11, −0.45)	0

#### 3.8.3 Rank of SUCRA

The order of SUCRA for the five prescriptions in adjusting LH was CFDTD+WM (SUCRA = 80.3%) > MRSSK+WM (SUCRA = 77.2%)>PIKDP+WM (SUCRA = 56.6%) > HSP + WM (SUCRA = 51.8%) > QHZMD+WM (SUCRA = 32.1%) > WM (SUCRA = 2.0%) (see Figure 4).

### 3.9 T

#### 3.9.1 Meta-analysis

Nine (14, 16, 17, 21–26) RCTs reported T, involving five prescriptions. The heterogeneity test showed that there was a large heterogeneity among the studies (*Ι^2^* = 63.6%, *p* = 0.005), so the random effect model was used to combine the effect size. The analysis results showed that ISRKRPP+WM in the treatment of obese PCOS was better than that of simple WM. The difference exhibited statistical significance (MD = −0.88, 95% CI [−1.14, −0.61], *p* < 0.05). Excluding literature one by one or making subgroup analyses according to BMI before treatment, treatment course, specific Chinese medicine prescription category of the experimental group, and type of WM used, no significant change in heterogeneity was found.

#### 3.9.2 Network meta-analysis

The results showed that PIKDP+WM (MD = −0.54.95% CI [−0.94, −0.14]), CFDTD+WM (MD = -0.46.95% CI [−0.82, −0.09]), MRSSK+WM (MD = −0.65.95% CI [−0.96, −0.35]), and HSP + WM (MD = −1.39.95% CI [−2.01, −0.77]) were statistically significant differences in reducing T, which were superior to WM treatment alone. Pairwise comparison of the interventions showed that HSP + WM (MD = −1.00, 95% CI [−1.80, −0.20]) was outperformed to QHZMD+WM; HSP + WM (MD = −0.85, 95% CI [−1.59, −0.11]) was superior to PIKDP+WM; HSP + WM (MD = −0.93.95% CI [−1.65, −0.21]) was superior to CFDTD+WM; HSP + WM (MD = −0.74.95% CI [−1.43, −0.05]) was superior to MRSSK+WM, and the difference of each of the above results was statistically significant. There was no statistically significant difference in pairwise comparison among the other interventions (see Table 6).

**Table 6:** Network meta-analysis of T.

Intervention	MD (95% CI)					
	PIKDP+WM	CFDTD+WM	MRSSK+WM	HSP + WM	QHZMD+WM	WM
PIKDP+WM	0					
CFDTD+WM	−0.08 (−0.62, 0.46)	0				
MRSSK+WM	0.11 (−0.39, 0.61)	0.20 (−0.28, 0.67)	0			
HSP + WM	0.85 (0.11, 1.59)	0.93 (0.21, 1.65)	0.74 (0.05, 1.43)	0		
QHZMD+WM	−0.15 (−0.80, 0.49)	−0.07 (−0.69, 0.55)	−0.27 (−0.86, 0.32)	−1.00 (−1.80, −0.20)	0	
WM	−0.54 (−0.94, −0.14)	−0.46 (−0.82, −0.09)	−0.65 (−0.96, −0.35)	−1.39 (−2.01, −0.77)	−0.39 (−0.89, 0.12)	0

#### 3.9.3 Rank of SUCRA

The order of SUCRA for the five prescriptions in reducing T was HSP + WM (SUCRA = 99.2%) > MRSSK+WM (SUCRA = 66.2%)>PIKDP+WM (SUCRA = 52.6%) > CFDTD+WM (SUCRA = 43.4%) > QHZMD+WM (SUCRA = 36.9%) > WM (SUCRA = 1.5%), as in Figure 4.

### 3.10 Adverse reaction

Six articles (14, 16, 18, 22, 25, 26) reported adverse reactions, which were analyzed descriptively due to inconsistent definitions of adverse reactions in different articles, preventing a combined comparison. Two articles (16, 22) reported no adverse reactions in either group. Liu et al. (14) reported two patients stopped metformin because of severe diarrhea in the control group; Huang et al. (18) reported two patients with nausea and vomiting, one patient with dyspepsia in the control group, two patients with nausea and vomiting, and two patients with intermittent diarrhea in the experimental group. Zhou et al. (25) reported mild nausea and abdominal distension in two patients from the experimental group, which were relieved with symptomatic treatment; Bai et al. (26) reported two patients in the control group and three in the experimental group experienced nausea and vomiting, but they improved within 30 min of rest without special treatment.

### 3.11 Publication bias

**Figure 5:**
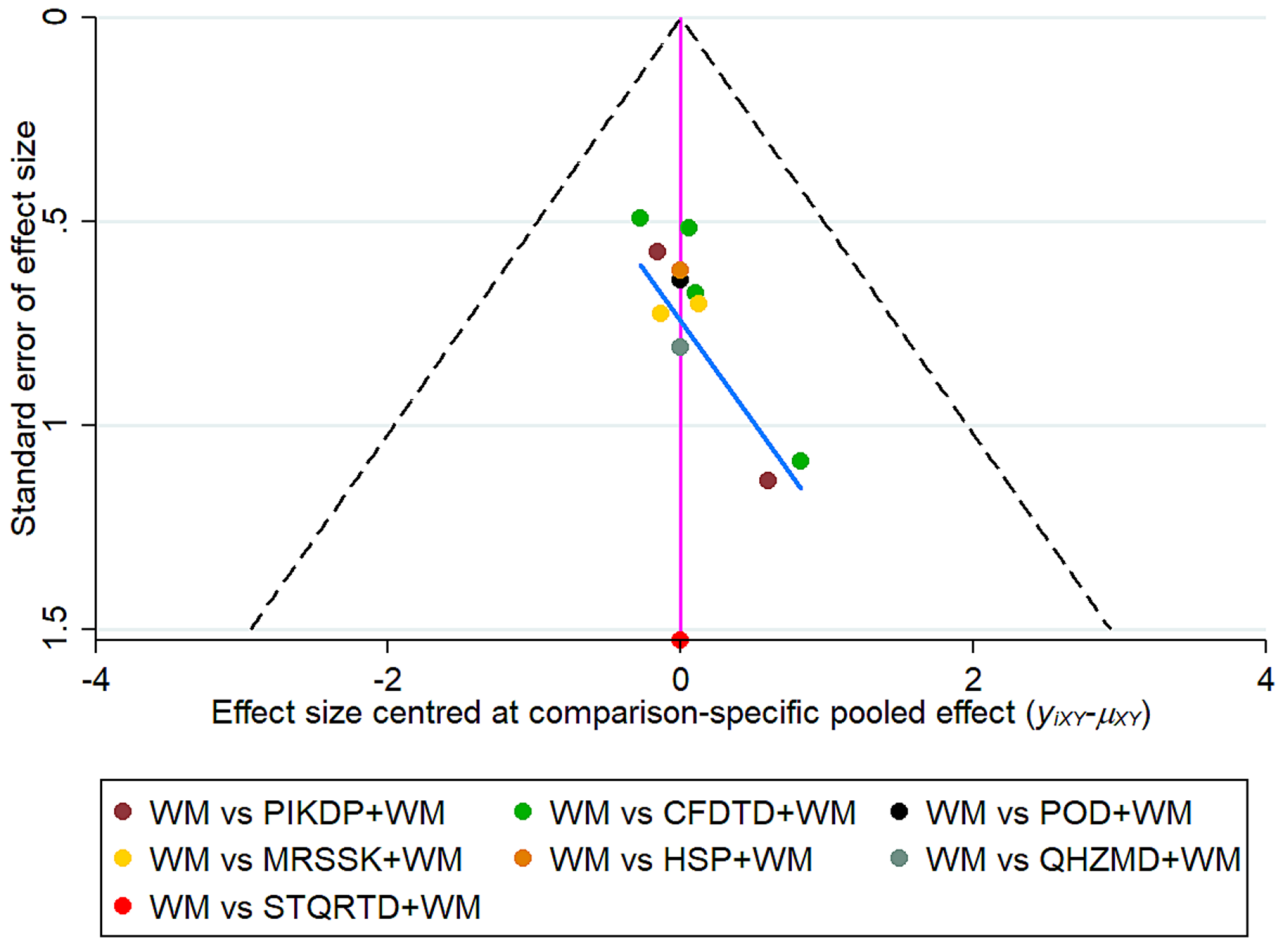
Comparison-correction funnel plot.

**Figure 6:**
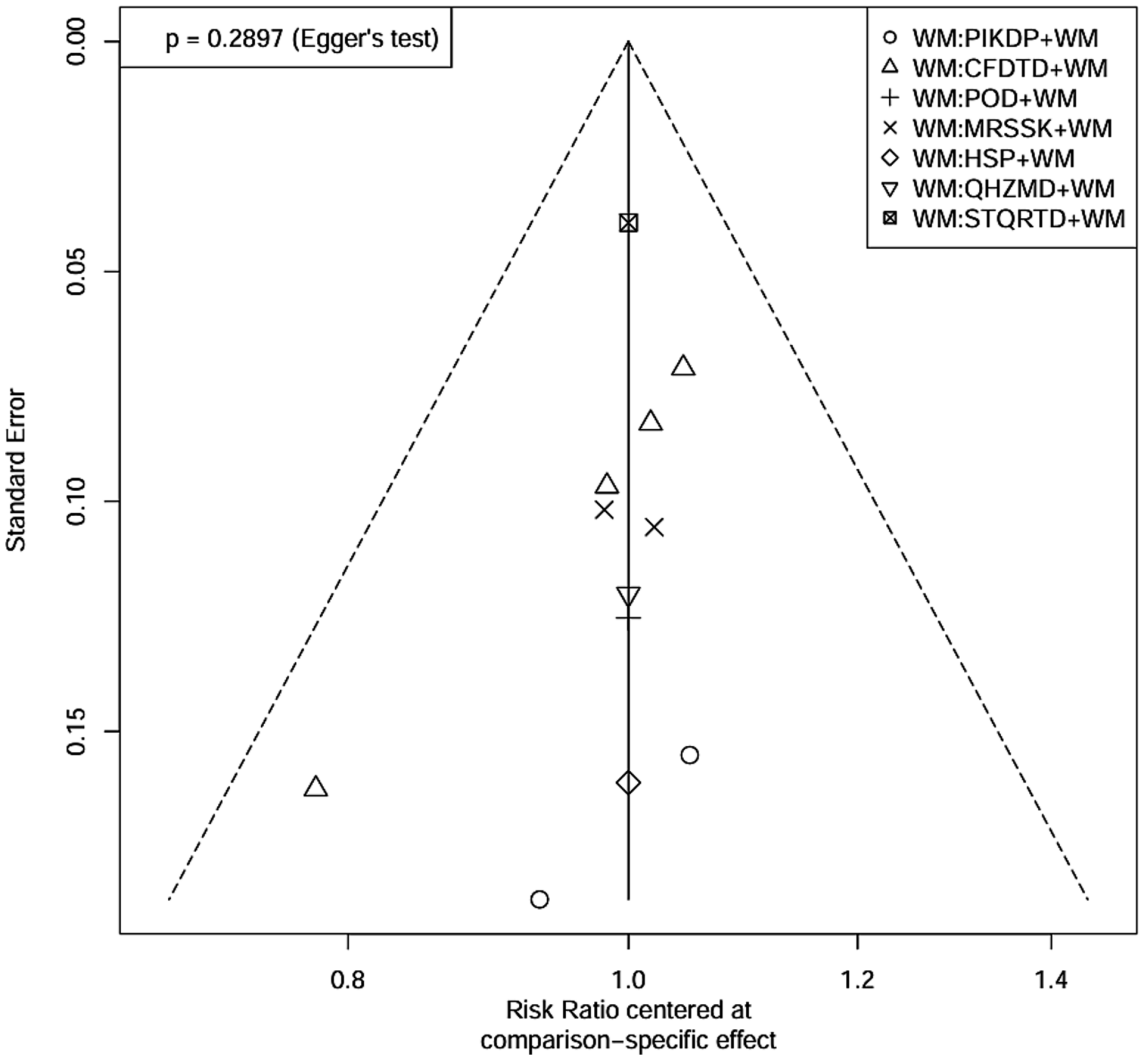
Egger’s test result.

Comparison-correction funnel plots were drawn to assess publication bias, represented by total clinical effectiveness. The results showed that most of the included studies were located on both sides of the pink zero line. In addition, a few studies showed a discrete asymmetric distribution, and the image showed a certain slope. This suggested the potential presence of publication bias, as in Figure 5. To further explore whether there was publication bias (28), an Egger’s test was conducted (29), as in Figure 6. The results indicated *p* = 0.2897, which was more than 0.05. Thus, no significant publication bias was detected, and the findings were robust.

### 3.12 Confidence of evidence

The confidence evidence grading results of the network meta-analysis showed that pairwise comparisons under all outcome measures were rated as moderate. These results indicated that they can provide certain support for the corresponding research conclusions, but high-quality research in the future still has the potential to change the current conclusions. It is suggested that when applying the relevant conclusions in this research, clinicians need to make decisions based on the individual conditions of patients and clinical experience. CINeMA results of clinical efficacy, BMI, HOMA-IR, LH, and T are shown in Tables 7–11.

**Table 7:** CINeMA results of clinical efficacy.

Comparison	Within-study bias	Reporting bias	Indirectness	Imprecision	Heterogeneity	Incoherence	Confidence rating
CFDTD and WM:WM	Some concerns	Undetected	No concerns	No concerns	No concerns	Major concerns	Moderate
HSP and WM:WM	Some concerns	Undetected	No concerns	Major concerns	No concerns	Major concerns	Moderate
MRSSK and WM:WM	Some concerns	Undetected	No concerns	No concerns	Major concerns	Major concerns	Moderate
PIKDP and WM:WM	Some concerns	Undetected	No concerns	No concerns	No concerns	Major concerns	Moderate
POD and WM:WM	Some concerns	Undetected	No concerns	No concerns	Major concerns	Major concerns	Moderate
QHZMD and WM:WM	Some concerns	Undetected	No concerns	No concerns	No concerns	Major concerns	Moderate
STQRTD and WM:WM	Some concerns	Undetected	No concerns	Major concerns	No concerns	Major concerns	Moderate
CFDTD and WM:HSP and WM	Some concerns	Undetected	No concerns	Major concerns	No concerns	Major concerns	Moderate
CFDTD and WM:MRSSK and WM	Some concerns	Undetected	No concerns	Major concerns	No concerns	Major concerns	Moderate
CFDTD and WM:PIKDP and WM	Some concerns	Undetected	No concerns	Major concerns	No concerns	Major concerns	Moderate
CFDTD and WM:POD and WM	Some concerns	Undetected	No concerns	Major concerns	No concerns	Major concerns	Moderate
CFDTD and WM:QHZMD and WM	Some concerns	Undetected	No concerns	Major concerns	No concerns	Major concerns	Moderate
CFDTD and WM:STQRTD and WM	Some concerns	Undetected	No concerns	No concerns	Major concerns	Major concerns	Moderate
HSP and WM:MRSSK and WM	Some concerns	Undetected	No concerns	Major concerns	No concerns	Major concerns	Moderate
HSP and WM:PIKDP and WM	Some concerns	Undetected	No concerns	Major concerns	No concerns	Major concerns	Moderate
HSP and WM:POD and WM	Some concerns	Undetected	No concerns	Major concerns	No concerns	Major concerns	Moderate
HSP and WM:QHZMD and WM	Some concerns	Undetected	No concerns	Major concerns	No concerns	Major concerns	Moderate
HSP and WM:STQRTD and WM	Some concerns	Undetected	No concerns	Major concerns	No concerns	Major concerns	Moderate
MRSSK and WM:PIKDP and WM	Some concerns	Undetected	No concerns	Major concerns	No concerns	Major concerns	Moderate
MRSSK and WM:POD and WM	Some concerns	Undetected	No concerns	Major concerns	No concerns	Major concerns	Moderate
MRSSK and WM:QHZMD and WM	Some concerns	Undetected	No concerns	Major concerns	No concerns	Major concerns	Moderate
MRSSK and WM:STQRTD and WM	Some concerns	Undetected	No concerns	Major concerns	No concerns	Major concerns	Moderate
PIKDP and WM:POD and WM	Some concerns	Undetected	No concerns	Major concerns	No concerns	Major concerns	Moderate
PIKDP and WM:QHZMD and WM	Some concerns	Undetected	No concerns	Major concerns	No concerns	Major concerns	Moderate
PIKDP and WM:STQRTD and WM	Some concerns	Undetected	No concerns	No concerns	Major concerns	Major concerns	Moderate
POD and WM:QHZMD and WM	Some concerns	Undetected	No concerns	Major concerns	No concerns	Major concerns	Moderate
POD and WM:STQRTD and WM	Some concerns	Undetected	No concerns	Major concerns	No concerns	Major concerns	Moderate
QHZMD and WM:STQRTD and WM	Some concerns	Undetected	No concerns	No concerns	Major concerns	Major concerns	Moderate

Yellow, Some concerns; Green, Undetected, No concerns; Red, Major concerns.

**Table 8:** CINeMA results of BMI.

Comparison	Within-study bias	Reporting bias	Indirectness	Imprecision	Heterogeneity	Incoherence	Confidence rating
CFDTD and WM:WM	Some concerns	Undetected	No concerns	No concerns	Major concerns	Major concerns	Moderate
HSP and WM:WM	Some concerns	Undetected	No concerns	No concerns	Major concerns	Major concerns	Moderate
MRSSK and WM:WM	Some concerns	Undetected	No concerns	No concerns	Major concerns	Major concerns	Moderate
PIKDP and WM:WM	Some concerns	Undetected	No concerns	No concerns	Major concerns	Major concerns	Moderate
STQRTD and WM:WM	Some concerns	Undetected	No concerns	No concerns	Major concerns	Major concerns	Moderate
CFDTD and WM:HSP and WM	Some concerns	Undetected	No concerns	Major concerns	No concerns	Major concerns	Moderate
CFDTD and WM:MRSSK and WM	Some concerns	Undetected	No concerns	No concerns	Major concerns	Major concerns	Moderate
CFDTD and WM:PIKDP and WM	Some concerns	Undetected	No concerns	No concerns	Major concerns	Major concerns	Moderate
CFDTD and WM:STQRTD and WM	Some concerns	Undetected	No concerns	No concerns	Major concerns	Major concerns	Moderate
HSP and WM:MRSSK and WM	Some concerns	Undetected	No concerns	No concerns	Major concerns	Major concerns	Moderate
HSP and WM:PIKDP and WM	Some concerns	Undetected	No concerns	No concerns	Major concerns	Major concerns	Moderate
HSP and WM:STQRTD and WM	Some concerns	Undetected	No concerns	No concerns	Major concerns	Major concerns	Moderate
MRSSK and WM:PIKDP and WM	Some concerns	Undetected	No concerns	Major concerns	No concerns	Major concerns	Moderate
MRSSK and WM:STQRTD and WM	Some concerns	Undetected	No concerns	Major concerns	No concerns	Major concerns	Moderate
PIKDP and WM:STQRTD and WM	Some concerns	Undetected	No concerns	Major concerns	No concerns	Major concerns	Moderate

Yellow, Some concerns; Green, Undetected, No concerns; Red, Major concerns.

**Table 9:** CINeMA results of HOMA-IR.

Comparison	Within-study bias	Reporting bias	Indirectness	Imprecision	Heterogeneity	Incoherence	Confidence rating
HSP and WM:WM	Some concerns	Undetected	No concerns	Major concerns	No concerns	Major concerns	Moderate
MRSSK and WM:WM	Some concerns	Undetected	No concerns	No concerns	Major concerns	Major concerns	Moderate
PIKDP and WM:WM	Some concerns	Undetected	No concerns	No concerns	Major concerns	Major concerns	Moderate
QHZMD and WM:WM	Some concerns	Undetected	No concerns	Major concerns	No concerns	Major concerns	Moderate
HSP and WM:MRSSK and WM	Some concerns	Undetected	No concerns	Major concerns	No concerns	Major concerns	Moderate
HSP and WM:PIKDP and WM	Some concerns	Undetected	No concerns	Major concerns	No concerns	Major concerns	Moderate
HSP and WM:QHZMD and WM	Some concerns	Undetected	No concerns	Major concerns	No concerns	Major concerns	Moderate
MRSSK and WM:PIKDP and WM	Some concerns	Undetected	No concerns	Major concerns	No concerns	Major concerns	Moderate
MRSSK and WM:QHZMD and WM	Some concerns	Undetected	No concerns	Major concerns	No concerns	Major concerns	Moderate
PIKDP and WM:QHZMD and WM	Some concerns	Undetected	No concerns	Major concerns	No concerns	Major concerns	Moderate

Yellow, Some concerns; Green, Undetected, No concerns; Red, Major concerns.

**Table 10:** CINeMA results of LH.

Comparison	Within-study bias	Reporting bias	Indirectness	Imprecision	Heterogeneity	Incoherence	Confidence rating
CFDTD and WM:WM	Some concerns	Undetected	No concerns	No concerns	Major concerns	Major concerns	Moderate
HSP and WM:WM	Some concerns	Undetected	No concerns	Major concerns	No concerns	Major concerns	Moderate
MRSSK and WM:WM	Some concerns	Undetected	No concerns	No concerns	Major concerns	Major concerns	Moderate
PIKDP and WM:WM	Some concerns	Undetected	No concerns	No concerns	Major concerns	Major concerns	Moderate
QHZMD and WM:WM	Some concerns	Undetected	No concerns	No concerns	Major concerns	Major concerns	Moderate
CFDTD and WM:HSP and WM	Some concerns	Undetected	No concerns	Major concerns	No concerns	Major concerns	Moderate
CFDTD and WM:MRSSK and WM	Some concerns	Undetected	No concerns	Major concerns	No concerns	Major concerns	Moderate
CFDTD and WM:PIKDP and WM	Some concerns	Undetected	No concerns	Major concerns	No concerns	Major concerns	Moderate
CFDTD and WM:QHZMD and WM	Some concerns	Undetected	No concerns	Major concerns	No concerns	Major concerns	Moderate
HSP and WM:MRSSK and WM	Some concerns	Undetected	No concerns	Major concerns	No concerns	Major concerns	Moderate
HSP and WM:PIKDP and WM	Some concerns	Undetected	No concerns	Major concerns	No concerns	Major concerns	Moderate
HSP and WM:QHZMD and WM	Some concerns	Undetected	No concerns	Major concerns	No concerns	Major concerns	Moderate
MRSSK and WM:PIKDP and WM	Some concerns	Undetected	No concerns	Major concerns	No concerns	Major concerns	Moderate
MRSSK and WM:QHZMD and WM	Some concerns	Undetected	No concerns	No concerns	Major concerns	Major concerns	Moderate
PIKDP and WM:QHZMD and WM	Some concerns	Undetected	No concerns	Major concerns	No concerns	Major concerns	Moderate

Yellow, Some concerns; Green, Undetected, No concerns; Red, Major concerns.

**Table 11:** CINeMA results of T.

Comparison	Within-study bias	Reporting bias	Indirectness	Imprecision	Heterogeneity	Incoherence	Confidence rating
CFDTD and WM:WM	Some concerns	Undetected	No concerns	No concerns	Major concerns	Major concerns	Moderate
HSP and WM:WM	Some concerns	Undetected	No concerns	No concerns	No concerns	Major concerns	Moderate
MRSSK and WM:WM	Some concerns	Undetected	No concerns	No concerns	Major concerns	Major concerns	Moderate
PIKDP and WM:WM	Some concerns	Undetected	No concerns	No concerns	Major concerns	Major concerns	Moderate
QHZMD and WM:WM	Some concerns	Undetected	No concerns	Major concerns	No concerns	Major concerns	Moderate
CFDTD and WM:HSP and WM	Some concerns	Undetected	No concerns	No concerns	Major concerns	Major concerns	Moderate
CFDTD and WM:MRSSK and WM	Some concerns	Undetected	No concerns	Major concerns	No concerns	Major concerns	Moderate
CFDTD and WM:PIKDP and WM	Some concerns	Undetected	No concerns	Major concerns	No concerns	Major concerns	Moderate
CFDTD and WM:QHZMD and WM	Some concerns	Undetected	No concerns	Major concerns	No concerns	Major concerns	Moderate
HSP and WM:MRSSK and WM	Some concerns	Undetected	No concerns	No concerns	Major concerns	Major concerns	Moderate
HSP and WM:PIKDP and WM	Some concerns	Undetected	No concerns	No concerns	Major concerns	Major concerns	Moderate
HSP and WM:QHZMD and WM	Some concerns	Undetected	No concerns	No concerns	Major concerns	Major concerns	Moderate
MRSSK and WM:PIKDP and WM	Some concerns	Undetected	No concerns	Major concerns	No concerns	Major concerns	Moderate
MRSSK and WM:QHZMD and WM	Some concerns	Undetected	No concerns	Major concerns	No concerns	Major concerns	Moderate
PIKDP and WM:QHZMD and WM	Some concerns	Undetected	No concerns	Major concerns	No concerns	Major concerns	Moderate

Yellow, Some concerns; Green, Undetected, No concerns; Red, Major concerns.

## 4 Summary of herbal prescription regularity

### 4.1 Data source

The corresponding prescriptions were identified from the 14 articles included in the network meta-analysis mentioned above.

### 4.2 Database establishment and statistical methods

The treatment prescriptions included in the literature were entered into Excel, with fields for the name of the prescription, herb names. The names of herbs were based on the Pharmacopeia of the People’s Republic of China (2020 edition) (30) and the Science of Chinese Materia Medica (31) to unify the different names of the same herbs. In addition, based on these two references, the efficacy of each herb was classified and the database was established. Using the Excel data, the frequency, properties (including four qi, five flavors, and meridian), and efficacy of the included herb were analyzed. Cluster analysis was performed for high-frequency herbs, and the results were presented through the tree diagram. Based on R language, the relevant prescription information in Excel was processed in data format, and the association rules were analyzed using the Apriori function.

### 4.3 Results

#### 4.3.1 Herb use frequency

The composition of the formulas in the 14 articles is shown in Table 12. Frequency analysis of these prescriptions revealed a total of 53 herbs. The top 30 herbs are shown in Table 13.

**Table 12:** Herb’s composition of the included literature.

Literature resources	Composition of Chinese medicine
Liu et al. (14)	Fluorite fluoritum; Epimedium; Citrus; *Pinellia ternata*; Poria cocos; Rhizoma atractylodis; *Achyranthes bidentata*; Red peony root; Angelica sinensis; Salviae miltiorrhizae; Rhizoma chuanxiong; Rhizoma cyperi; Hawthorn
Lu et al. (15)	Angelica sinensis; Prepared rehmannia root; *Curculigo orchioides*; clove; Epimedium; *Ligustrum lucidum* ait; turtle shell; Salviae miltiorrhizae; Tree peony bark; Rhizoma cyperi; *Acorus gramineus*; Poria cocos; Rhizoma atractylodis; Atractylodes macrocephala Koidz; *Pinellia ternata*; malt; Medicated leaven; Hawthorn
Hou et al. (16)	Epimedium; Fluorite fluoritum; Morinda officinalis; Cornu cervi degelatinatum; Semen cuscutae; Rhizoma atractylodis; Poria cocos; citrus; *Pinellia ternata*; Spina gleditsiae; *Achyranthes bidentata*; Angelica sinensis; Rhizoma cyperi; Hawthorn; prickly ash; Radix glycyrrhiza
Guo et al. (17)	Rhizoma atractylodis; *Pinellia ternata*; citrus; Rhizoma cyperi; Radix astragali; Poria cocos; Epimedium; *Acorus gramineus*; Angelica sinensis; Spina gleditsiae; Salviae miltiorrhizae
Huang et al. (18)	Rhizoma atractylodis; Rhizoma cyperi; citrus; *Pinellia ternata*; Spina gleditsiae; Angelica sinensis; Rhizoma chuanxiong; *Acorus gramineus*; lotus leaf; yam; Poria cocos; human placenta; Radix astragali; Salviae miltiorrhizae; Cornu cervi degelatinatum; Fluorite fluoritum; Fructus amomi
Shan et al. (19)	Radix astragali; yam; Poria cocos; Salviae miltiorrhizae; Epimedium; Spina gleditsiae; Rhizoma cyperi; *Pinellia ternata*; *Acorus gramineus*; Rhizoma atractylodis; angelica sinensis; citrus
Zeng et al. (20)	Angelica sinensis; Semen cuscutae; Atractylodes macrocephala Koidz; Rhizoma chuanxiong; Tree peony bark; Salviae miltiorrhizae; Semen coicis; Dipsacus; Red peony root; Rhizoma atractylodis; Poria cocos
Zhang et al. (21)	Radix codonopsis; Atractylodes macrocephala Koidz; Poria cocos; Fructus psoraleae; Prepared rehmannia root; yam; Dipsacus; Epimedium; Semen cuscutae; Leonurus heterophyllus; Red peony root; *Pinellia ternata*; citrus; Red yeast rice; *Acorus gramineus*; Radix glycyrrhizae
Li et al. (22)	Prepared rehmannia root; Radix codonopsis; Fructus lycii; Epimedium; Cistanche; Angelica sinensis; Atractylodes Macrocephala Koidz; Poria cocos; India mustard seed; citrus; Cornu cervi degelatinatum; Pericarpium citri reticulatae viride; Radix glycyrrhizae
Liu et al. (23)	Radix astragali; Red peony root; Angelica sinensis; Poria cocos; Semen cuscutae; Epimedium; Cistanche
Liang et al. (24)	Radix astragali; yam; Rhizoma atractylodis; Epimedium; Salviae miltiorrhizae; *Pinellia ternata*; Spina gleditsiae; Angelica sinensis; Rhizoma cyperi; Bile arisaema; citrus
Zhou et al. (25)	Lotus leaf; Radix astragali; Semen cassiae; *Polygonum multiflorum*; yam; Exocarpium benincasae; *Acorus gramineus*
Bai et al. (26)	Radix astragali; Prepared rehmannia root; Polygonatum kingianum; Coptidis; Pseudo-ginseng; Rhizoma atractylodis; Gynostemma pentaphyllum
Wang et al. (27)	Radix astragali; Atractylodes macrocephala koidz; Rhizoma atractylodis; Lotus leaf; Exocarpium benincasae; Hawthorn; Fructus lycii; yam; Semen coicis; Ground beeltle; *Pinellia ternata*; *Acorus gramineus*

**Table 13:** Count and percentage of top 30 herbs.

Herb	Count	Percentage %
Rhizoma atractylodis	10	71.43%
Poria cocos	10	71.43%
Angelica sinensis	10	71.43%
*Pinellia ternata*	9	64.29%
Epimedium	9	64.29%
Citrus	8	57.14%
Radix astragali	8	57.14%
*Acorus gramineus*	7	50.00%
Salviae miltiorrhizae	7	50.00%
Rhizoma cyperi	7	50.00%
Yam	6	42.86%
Atractylodes macrocephala koidz	5	35.71%
Spina gleditsiae	5	35.71%
Semen cuscutae	4	28.57%
Red peony root	4	28.57%
Prepared rehmannia root	4	28.57%
Hawthorn	4	28.57%
Fluorite fluoritum	3	21.43%
Cornu cervi degelatinatum	3	21.43%
Radix glycyrrhizae	3	21.43%
Lotus leaf	3	21.43%
Rhizoma chuanxiong	3	21.43%
Tree peony bark	2	14.29%
Cistanche	2	14.29%
Radix codonopsis	2	14.29%
*Achyranthes bidentata*	2	14.29%
Exocarpium benincasae	2	14.29%
Semen coicis	2	14.29%
Dipsacus	2	14.29%
Fructus lycii	2	14.29%

#### 4.3.2 Drug efficacy, four qi, five flavors, and meridians statistics

After analysis, four qi of top three were warm, neutral, or slightly warm, as detailed in Table 14; five flavors of top three were sweet, pungent, bitter, as detailed in Table 15; the top three belonging meridians were spleen, liver, and kidney, as detailed in Table 16; drugs can be divided into 11 categories according to their efficacy and the category with the largest proportion is tonic drugs, as detailed in Table 17.

**Table 14:** Count and percentage of the four qi.

Four qi	Count	Percentage %
Warm	83	48.54%
Neutral	41	23.98%
Slight warm	20	11.70%
Slight cold	16	9.36%
Cool	7	4.09%
Cold	2	1.17%
Hot	1	0.58%
Severe cold	0	0.00%
Severe hot	0	0.00%

**Table 15:** Count and percentage of five flavors.

Five flavors	Count	Percentage %
Sweet	88	51.46%
Pungent	87	50.88%
Bitter	61	35.67%
Light	12	7.02%
Salty	9	5.26%
Slight sweet	8	4.68%
Slight bitter	8	4.68%
Sour	6	3.51%
Astringent	4	2.34%
Slight pungent	1	0.58%
Slight salty	0	0.00%
Slight sour	0	0.00%
Slight astringent	0	0.00%

**Table 16:** Count and percentage of drug meridian.

Meridian	Count	Percentage %
Foot greater yin spleen meridian	104	60.82%
Foot reverting yin liver meridian	94	54.97%
Foot lesser yin kidney meridian	60	35.09%
Foot yang brightness stomach meridian	56	32.75%
Hand great yin lung meridian	56	32.75%
Hand lesser yin heart meridian	45	26.32%
Hand lesser yang triple energizer meridian	7	4.09%
Hand yang brightness large intestine meridian	5	2.92%
Foot lesser yang gallbladder meridian	5	2.92%
Hand reverting yin pericardium meridian	4	2.34%
Hand greater yang small intestine meridian	2	1.17%
Foot greater yang bladder meridian	1	0.58%

**Table 17:** Count and percentage of drug efficacy.

Efficacy category	Count	Percentage %
Tonifying medicinal	72	42.11%
Qi tonifying medicinal	25	14.62%
Yang-tonifying medicinal	27	15.79%
Yin-tonifying medicinal	5	2.92%
Blood-tonifying medicinal	15	8.77%
Heat-clearing medicinal	16	9.36%
Heat-clearing and detoxicating medicinal	5	2.92%
Heat-clearing and fire-purging medicinal	4	2.34%
Heat-clearing and dampness-drying medicinal	1	0.58%
Heat-clearing and blood cooling medicinal	6	3.51%
Qi-regulating medicinal	16	9.36%
Blood-activating and Stasis-resolving Medicinal	15	8.77%
Edema-alleviating diuretic	14	8.19%
Damp-resolving medicinal	11	6.43%
Phlegm-resolving, antitussive and antiasthmatic medicinal	11	6.43%
Warming cold phlegm medicinal	10	5.85%
Clearing heat phlegm medicinal	1	0.58%
Resuscitative medicinal	7	4.09%
Digestant medicinal	6	3.51%
Interior-warming medicinal	2	1.17%
Hemostatic medicinal	1	0.58%
Stasis-resolving hemostatic	1	1.00%

#### 4.3.3 Cluster analysis

**Figure 7:**
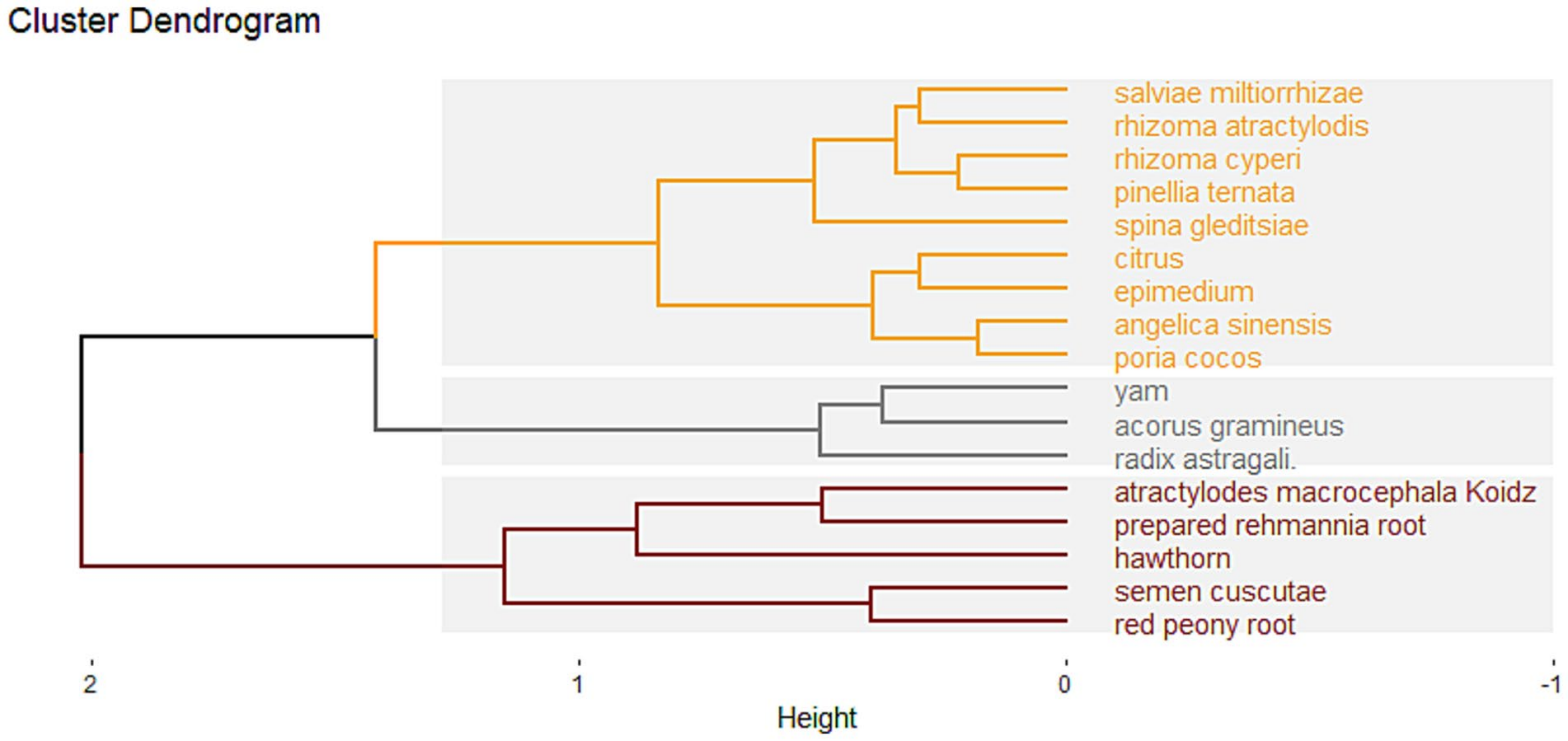
Cluster analysis diagram.

Cluster analysis was performed for the top 17 high-frequency herbs in the prescription, as shown in Figure 7.

Three cluster groups were formed using grouping distance > 1.25 as the boundary. Cluster 1: Epimedium, Poria cocos, Angelica sinensis, Citrus, Spina gleditsiae, Salviae miltiorrhizae, Rhizoma atractylodis, *Pinellia ternata*, and Rhizoma cyperi; Cluster 2: Radix astragali, *Acorus gramineus*, and Yam; Cluster 3: Hawthorn, Semen cuscutae, Red peony root, Atractylodes macrocephala koidz, and prepared rehmannia root.

#### 4.3.4 Association rule analysis

**Figure 8:**
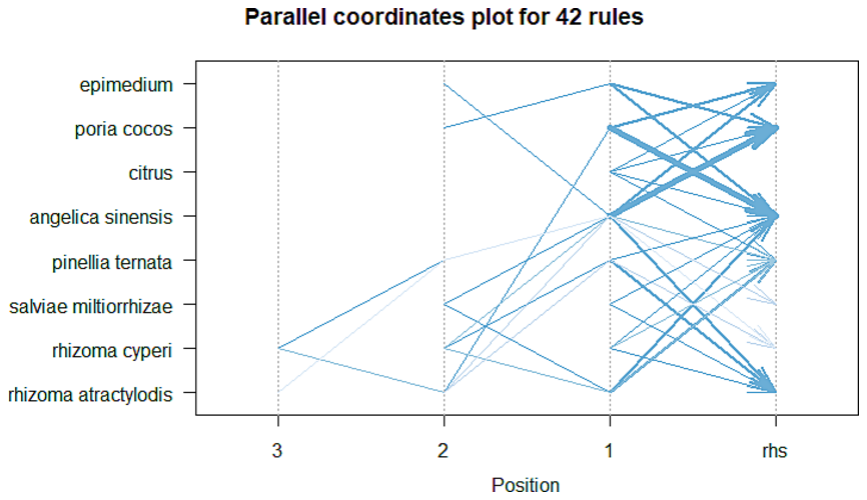
Association rule network diagram.

**Figure 9:**
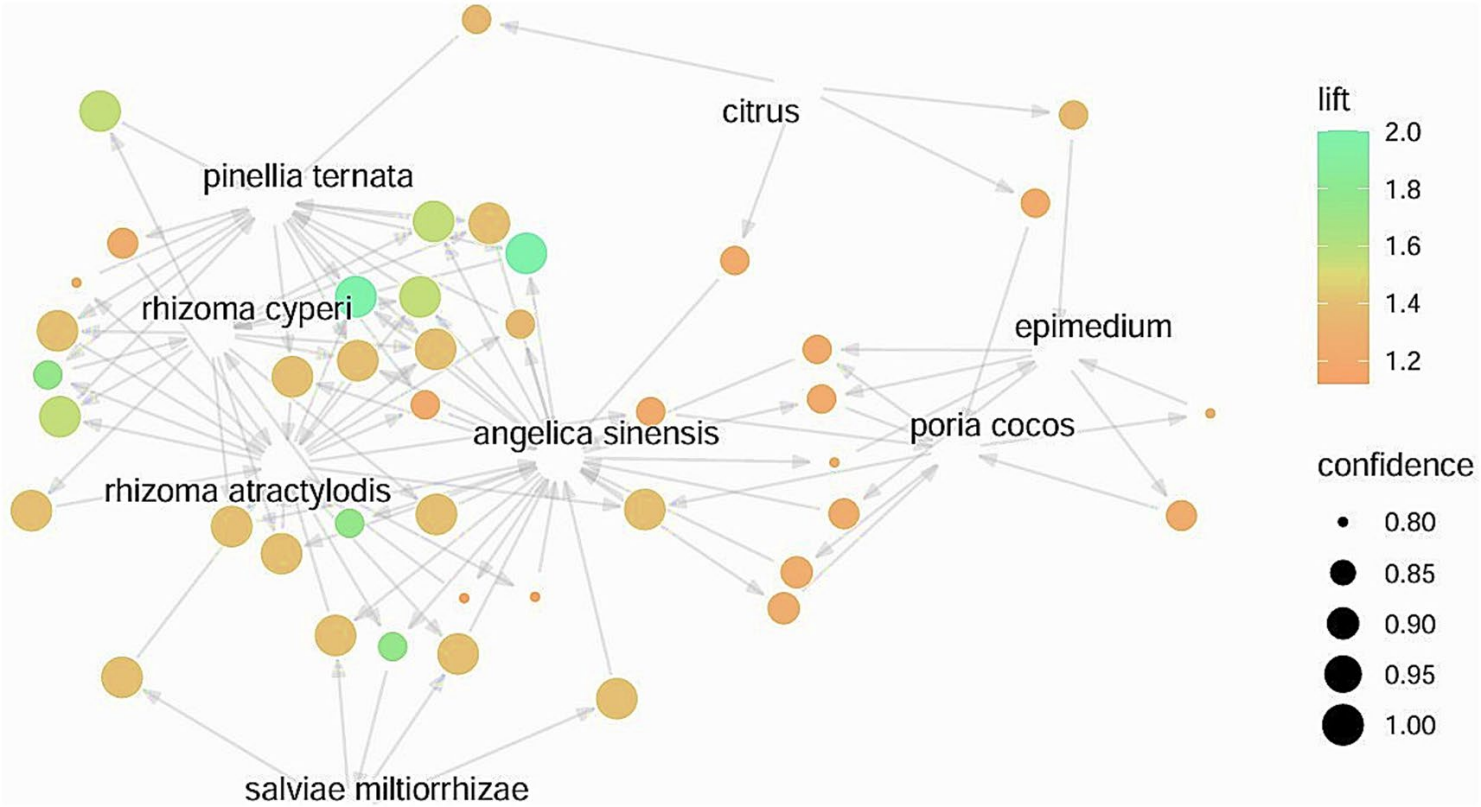
Association rule parallel coordinate diagram.

By setting a minimum support level of 50% and a confidence level of 80%, the herbs were analyzed using association rules, resulting in 42 pairs of associated herbs. The top 15 pairs, ranked by support degree, are shown in Table 18. The network diagram of the association rules is plotted in Figure 8. The parallel coordinate diagram of the association rules is plotted in Figure 9.

**Table 18:** Ranking table of association rules.

Number	Lhs	Rhs	Support %	Confidence %	Lift
1	Angelica sinensis	Poria cocos	64.29%	90%	1.26
2	Poria cocos	Angelica sinensis	64.29%	90%	1.26
3	Epimedium	Angelica sinensis	57.14%	89%	1.24
4	Angelica sinensis	Epimedium	57.14%	80%	1.24
5	Epimedium	Poria cocos	57.14%	89%	1.24
6	Poria cocos	Epimedium	57.14%	80%	1.24
7	*Pinellia ternata*	Rhizoma atractylodis	57.14%	89%	1.24
8	Rhizoma atractylodis	*Pinellia ternata*	57.14%	80%	1.24
9	Rhizoma atractylodis	Angelica sinensis	57.14%	80%	1.12
10	Angelica sinensis	Rhizoma atractylodis	57.14%	80%	1.12
11	Salviae miltiorrhizae	Rhizoma atractylodis	50.00%	100%	1.40
12	Salviae miltiorrhizae	Angelica sinensis	50.00%	100%	1.40
13	Rhizoma cyperi	*Pinellia ternata*	50.00%	100%	1.56
14	Rhizoma cyperi	Rhizoma atractylodis	50.00%	100%	1.40
15	Rhizoma cyperi	Angelica sinensis	50.00%	100%	1.40

## 5 Discussion

PCOS is a reproductive endocrine disease characterized by menstrual disorders, infertility, and obesity. Approximately 80% of PCOS patients have abdominal/visceral obesity, which is closely related to pathological conditions such as IR, HA, and chronic inflammation. These pathologies interact, resulting in abnormalities in the hypothalamic–pituitary–ovarian axis and metabolic disturbances, which adversely affect female reproductive health and overall metabolic levels (32). Obese PCOS patients frequently present with IR, so the current Western medical treatment mainly uses insulin sensitizers. Some studies suggest that metformin has unique clinical advantages as it not only helps patients lose body weight but also positively impacts female reproductive health by improving ovulation rate, pregnancy rate, and endometrial tolerance (33). In addition, hormone replacement drugs, ovulation-stimulating drugs, and laparoscopic surgery are also available. However, these treatments may lead to adverse reactions such as nausea, vomiting, diarrhea, liver and kidney dysfunction, and ovarian hyperstimulation (34, 35).

TCM links the occurrence of PCOS to a disorder in the “kidney – reproductive essence – Chong and Ren meridian– Uterus.” The pathogenesis is characterized by kidney deficiency, spleen weakness, and liver qi stagnation, causing phlegm, turbidity, and blood stasis to accumulate in the uterus, which in turn results in reproductive system dysfunction. The Bamboo and Fountain Born Female Science Collection recorded: “late menstruation, purple and less… this is due to kidney depletion and water drying…, known as kidney deficiency.” The kidney and bladder are externally and internally connected. The kidney qi is weak, and the bladder is also weak, resulting in metabolic disturbances in the lower jiao, with water, qi, and blood obstructed, leading to “late menstruation, dark-colored and scanty menstrual flow,” even amenorrhea and infertility in severe cases (36). The Secret Book of the Orchid Chamber - Women’s Gate said: “A woman’s spleen and stomach have been weak for a long time… suffer from qi and blood deficiency, causing menstruation to cease.” Spleen dysfunction impairs the production of menstrual blood, disrupts follicular development, and obstructs ovulation (37). The Essential of Women′s Diseases (38) said: “Fat white women, delayed menstruation and amenorrhea, are often caused by phlegm-dampness and lipid membrane blockage.” Overweight/obese patients typically have excessive fat accumulation. The syndrome type of such patients in TCM is usually a damp phlegm pattern, due to phlegm dampness block, qi, and blood movement disorder, phlegm dampness, and blood stasis block hairy orifice, so there are acne, hirsutism, and other manifestations. Therefore, invigorating the spleen, replenishing the kidney, and resolving phlegm is an important treatment rule for obese PCOS.

A total of 14 RCTs involving 1,163 patients were included in this study, involving seven different prescriptions for invigorating the spleen, replenishing the kidney, and resolving phlegm. Direct meta-analysis showed that oral prescription for strengthening the spleen, invigorating the kidney, and resolving phlegm along with WM was superior to WM alone in improving all clinical indices in obese PCOS. This suggests that integrative Chinese and Western medicine has notable benefits for obese PCOS treatment. Network meta-analysis, used to indirectly compare various prescriptions, provided evidence to support the effectiveness comparison of different intervention measures (39). QHZMD+WM may be the best in improving overall clinical efficiency; CFDTD+WM may be the most effective in reducing BMI; MRSSK+WM may have an obvious impact on reducing HOMA-IR. CFDTD+WM may be the best in reducing LH; HSP + WM may be the most effective in lowering T. The above results can provide certain guidance for clinicians in selecting appropriate treatments for patients and help achieve better clinical outcomes.

Herbal Regularity analysis revealed that the most frequently used herbs for obese PCOS align with the theoretical characteristics of medications used to treat kidney-deficient, spleen-deficient, liver-depression, and phlegm-dampness block in PCOS patients. The top five most frequently used Chinese medicines were Rhizoma atractylodis, Poria cocos, Angelica sinensis, *Pinellia ternata*, and Epimedium. The drug pairs composed of the above five medicines, namely, Angelica sinensis-Poria cocos, Poria cocos-Angelica sinensis, Epimedium-Angelica sinensis, Angelica sinensis-Epimedium, and Epimedium-Poria cocos, were also among the top 5 highest-ranked in the association rule analysis, showing them as core combinations for treating obese PCOS. Modern pharmacological research has shown that the extract of Rhizoma atractylodis inhibits *α-glucosidase* activity (40), and its polysaccharide has strong interventional effects in alloxan-induced hyperglycemia mice by regulating blood glucose metabolism and promoting insulin secretion (41). In addition, Rhizoma atractylodis volatile oil, such as *β*-Eudesmol, increases the excretion of Na^+^, Cl^-^, while reducing the retention of water and sodium (42). Studies have shown that Poria cocos is water-insoluble polysaccharide, regulates intestinal flora, improves glucose and lipid metabolism, and reduces inflammatory response (43); it also promotes *PPARα* expression and inhibits the key regulators expression of *REBP-1*, *ACC1,* and *Fas* in fatty acid generation, thus improving hyperlipidemia and lipid accumulation (44). In addition, pachymic acid may protect ovarian damage function in PCOS rats by inhibiting the *Hippo-YAP* pathway (45), reducing inflammation, antagonizing androgen synthesis, and improving insulin sensitivity, thereby repairing the internal environment and inhibiting the programmed death of granulosa cells in the ovary (46). PCOS and related comorbidities are significantly associated with dysregulation of local oxidative stress in the ovary (47), while the anti-oxidant stress response is closely related to the expression of the *Keap1/Nrf2/HO-1* pathway in the body (48). A study showed that the ethanol extract of Angelica Sinensis could reduce the overexpression of Keap1 and meanwhile significantly enhance the expression of *Nrf2* and *HO-1* in PCOS model rats. This suggests that the ethanol extract of Angelica Sinensis could correct the imbalance in ovarian oxidative stress response in rats and avoid the ovarian tissues from further damage by accurately regulating the related expression of *Keap1/Nrf2/HO-1* (49). *Pinellia ternata*’s aqueous extract improves obesity by inhibiting intracellular lipid accumulation and increasing the expression levels of adipose triacylglycerol lipase, hormone-sensitive lipase, and autophagy-related gene 5 in mice to promote local lipolysis and fat phagocytosis (50). Epimedium improves ovarian function by inhibiting *Fas* overexpression, increasing the expression activity of estrogen and progesterone, and reducing *Bcl-2* expression (51). Its extract icariin can increase the serum AMH value, while significantly increasing the number of follicles, to exert the efficacy of ovarian protection (52); in addition, Icariin has significant antidiabetic activity, which may improve insulin resistance and pancreatic *β-cell* dysfunction (53).

Cluster analysis obtained three types of prescriptions. The first type of prescription has multiple functions: warming the kidney and strengthening the spleen, resolving phlegm and dispelling dampness, promoting blood circulation and removing blood stasis, and soothing the liver and regulating qi. It can comprehensively treat both superficial symptoms and root pathogenesis. The second type focuses on benefiting qi. The third type emphasizes strengthening the spleen and digestion, lowering lipids, and promoting pregnancy. All three kinds of prescriptions had the effects of invigorating the spleen, replenishing the kidney, and resolving phlegm. Combined with frequency analysis and association rule analysis, the first type of prescription may be the core prescription of obese PCOS. This prescription is based on the addition and subtraction of CFDTD, which strengthens the spleen, regulates qi, warms the middle, dries dampness, and resolves phlegm, with the addition of Epimedium to replenish the kidney and Spina gleditsiae to eliminate carbuncle and pus. It also learns from the concept of “Angelica sinensis and Red peony root Powder,” regulating qi and blood and removing dampness. Therefore, this prescription can schedule menstruation, significantly improve infertility, and reduce obesity or overweight. Modern pharmacology has confirmed that CFDTD has an overall regulatory effect in treating obese PCOS. By precisely regulating the gene transcription process of core targets, CFDTD influences estrogen signaling and corrects insulin resistance and the inflammatory microenvironment to optimize ovarian function. It also plays an important role in regulating interleukin immune signaling, adjusting lipid metabolism, and controlling the synthesis of steroid hormones (54, 55).

The existing studies on the method of invigorating the spleen, replenishing the kidney, and resolving phlegm in the treatment of obese PCOS only used a single prescription as an intervention to conduct a meta-analysis of the outcome indicators of patients. The sample size of such studies was small and insufficient to represent the therapeutic effect of all kinds of prescriptions in this method on obese PCOS disease. At the same time, due to the large number of prescriptions involved, there was a lack of direct comparison. Therefore, through network meta-analysis, this study verified that ISRKRPP combined with WM had better efficacy than WM alone in improving the related outcome indicators of obesity-type PCOS patients. Moreover, it analyzed the differences in the efficacy of different prescriptions in the treatment of obesity-type PCOS, summarized the rules of medication, and dug out the core combination of traditional Chinese medicine for the treatment of the disease. It has the advantages of verifying the conclusions of previous research, making up for the shortcomings of previous research, expanding the scope of research, and better guiding clinical treatment.

## 6 Limitations

There are some limitations. (1) The quality of the literature included was low, with simple randomization methods, and no allocation hiding or blind method, which might cause bias; (2) some studies had small sample sizes, potentially leading to small-sample effects; (3) difference of dosage and severity of patients’ disease may affect the treatment outcome; (4) differences in the conventional western treatments may affect the results to some extent; (5) the number of studies on different traditional Chinese medicines varied greatly, for example, there are fewer studies on HSP, STQRTD, POD, and QHZMD, leading to unstable efficacy ordering; (6) the lack of studies directly comparing interventions may affect the assessment of efficacy; (7) most studies did not specify the methods of measurement of endometrial thickness, number of follicles, and ovarian size, so statistical analyses were not performed; (8) the follow-up periods varied across studies, making it difficult to obtain consistent and representative data. Therefore, this study did not conduct a comprehensive and reasonable evaluation of the long-term efficacy.

## 7 Conclusion

In conclusion, combining prescription for invigorating the spleen, replenishing the kidney, and resolving phlegm with WM in the conventional treatment of obese PCOS may enhance the therapeutic efficacy, which is worthy of clinical promotion. In light of the study’s limitations, these conclusions need to be interpreted with caution. Future research should include large-scale, high-quality, multicenter clinical trials to further validate the findings.

## Data Availability

The original contributions presented in the study are included in the article/Supplementary material, further inquiries can be directed to the corresponding author.
