# Supplementary material for: Method of invigorating spleen and replenishing kidney and resolving phlegm for obesity-type polycystic ovary syndrome: a network meta-analysis and summary of herbal prescription regularity
Source: Front Med (Lausanne). 2025 Jul 28;12:1609131. doi: 10.3389/fmed.2025.1609131 (PMC12336145; doi:10.3389/fmed.2025.1609131)
Supplement: Supplementary file 1 [file Data_Sheet_1.docx]

| PubMed database | | |
| --- | --- | --- |
| Search number | Search Details | Results |
| 1 | "Obesity"[MeSH Terms] | 275917 |
| 2 | "Polycystic Ovary Syndrome"[MeSH Terms] | 19231 |
| 3 | "polycystic ovarian syndrome"[Title/Abstract] OR "polycystic ovary syndrome 1"[Title/Abstract] OR "sclerocystic ovarian degeneration"[Title/Abstract] OR "sclerocystic ovaries"[Title/Abstract] OR "sclerocystic ovary syndrome"[Title/Abstract] OR "stein leventhal syndrome"[Title/Abstract] | 5072 |
| 4 | "medicine, chinese traditional"[MeSH Terms] | 25584 |
| 5 | Chinese Medicine, Traditional[Title/Abstract] OR Chinese Traditional Medicine[Title/Abstract] OR Chung I Hsueh[Title/Abstract] OR Traditional Chinese Medicine[Title/Abstract] OR Traditional Medicine, Chinese[Title/Abstract] OR Traditional Tongue Assessment[Title/Abstract] OR Traditional Tongue Diagnosis[Title/Abstract] OR Zhong Yi Xue[Title/Abstract] OR TCM[Title/Abstract] | 44535 |
| 6 | "drugs, chinese herbal"[MeSH Terms] | 55733 |
| 7 | Chinese Drugs, Plant[Title/Abstract] OR Chinese Herbal Drugs[Title/Abstract] OR Plant Extracts, Chinese[Title/Abstract] OR Chinese Herbal[Title/Abstract] OR Tang[Title/Abstract] OR Fang[Title/Abstract] OR Prescription[Title/Abstract] | 136231 |
| 8 | "Randomized Controlled Trial"[Publication Type] | 623811 |
| 9 | "Randomized Controlled Trials as Topic"[MeSH Terms] OR "RCT"[Title/Abstract] OR "Randomly"[Title/Abstract] OR "Randomized"[Title/Abstract] OR "controlled clinical trial"[Title/Abstract] OR "clinical trial"[Title/Abstract] OR "clinical study"[Title/Abstract] | 1344588 |
| 10 | #2 OR #3 | 20982 |
| 11 | #4 OR #5 OR #6 OR #7 | 218266 |
| 12 | #8 OR #9 | 1516185 |
| 13 | #1 AND #10 AND #11 AND #12 | 5 |
| Embase datebase | | |
| 1 | 'randomized controlled trial':ab,ti OR rct:ab,ti OR randomly:ab,ti OR randomized:ab,ti OR 'controlled clinical trial':ab,ti OR 'clinical trial':ab,ti OR 'clinical study':ab,ti | 1787563 |
| 2 | 'ovary polycystic disease'/exp | 40791 |
| 3 | 'cystic ovary':ab,ti OR 'micropolycystic ovary':ab,ti OR 'multiple follicle cyst':ab,ti OR 'ovary polycystic syndrome':ab,ti OR 'ovary, micropolycystic':ab,ti OR 'ovary, polycystic':ab,ti OR 'polycystic ovarian disease':ab,ti OR 'polycystic ovary':ab,ti OR 'polycystic ovary disease':ab,ti OR 'polycystic ovary syndrome':ab,ti OR 'stein cohen leventhal syndrome':ab,ti OR 'stein leventhal disease':ab,ti OR 'stein leventhal syndrome':ab,ti OR 'syndrome stein leventhal':ab,ti OR 'ovary polycystic disease':ab,ti | 27366 |
| 4 | 'chinese medicine'/exp | 83465 |
| 5 | 'chinese herbal medicine':ti OR 'chinese traditional medicine':ti OR 'medicine, chinese traditional':ti OR 'traditional chinese medicine':ti OR 'chinese medicine':ti OR tang:ti OR fang:ti OR prescription:ti | 54306 |
| 6 | 'obesity'/exp | 726261 |
| 7 | 'adipose tissue hyperplasia':ab,ti OR 'adipositas':ab,ti OR 'adiposity':ab,ti OR 'alimentary obesity':ab,ti OR 'body weight, excess':ab,ti OR 'corpulency':ab,ti OR 'fat overload syndrome':ab,ti OR 'nutritional obesity':ab,ti OR 'obesitas':ab,ti OR 'overweight':ab,ti OR 'obesity':ab,ti | 570407 |
| 8 | #2 OR #3 | 42630 |
| 9 | #4 OR #5 | 120255 |
| 10 | #6 OR #7 | 825785 |
| 11 | #1 AND #8 AND #9 AND #10 | 13 |
| Cochrane Datebase | | |
| 1 | MeSH descriptor: [Polycystic Ovary Syndrome] explode all trees | 2171 |
| 2 | (Polycystic Ovarian Syndrome or Polycystic Ovary Syndrome 1 or Sclerocystic Ovarian Degeneration or Sclerocystic Ovaries or Sclerocystic Ovary Syndrome or Stein-Leventhal Syndrome):ti,ab,kw | 3706 |
| 3 | MeSH descriptor: [Medicine, Chinese Traditional] explode all trees | 1792 |
| 4 | (Chinese Medicine, Traditional or Chinese Traditional Medicine or Chung I Hsueh or Traditional Chinese Medicine or Traditional Medicine, Chinese or Traditional Tongue Assessment or Traditional Tongue Diagnosis or Zhong Yi Xue or TCM):ti,ab,kw | 15600 |
| 5 | MeSH descriptor: [Drugs, Chinese Herbal] explode all trees | 4673 |
| 6 | (Chinese Drugs, Plant or Chinese Herbal Drugs or Plant Extracts, Chinese or Chinese Herbal or Tang or Fang or Prescription):ti,ab,kw | 29810 |
| 7 | MeSH descriptor: [Randomized Controlled Trial] explode all trees | 37 |
| 8 | (RCT or Randomly or Randomized or Controlled Clinical Trial or Clinical Trial or Clinical Study):ti,ab,kw | 1576244 |
| 9 | MeSH descriptor: [Obesity] explode all trees | 21661 |
| 10 | #1 OR #2 | 4421 |
| 11 | #3 OR #4 | 15852 |
| 12 | #5 OR #6 | 29810 |
| 13 | #7 OR #8 | 1576244 |
| 14 | #11 OR #12 | 42125 |
| 15 | #9 AND #10 AND #13 AND #14 | 5 |
| Web Of Science database | | |
| 1 | TS=((Polycystic Ovary Syndrome OR Polycystic Ovarian Syndrome OR Polycystic Ovary Syndrome 1 OR Sclerocystic Ovarian Degeneration OR Sclerocystic Ovaries OR Sclerocystic Ovary Syndrome OR Stein-Leventhal Syndrome) AND obesity) | 4330 |
| 2 | TS=(Medicine, Chinese Traditional OR Chinese Medicine, Traditional OR Chinese Traditional Medicine OR Chung I Hsueh OR Traditional Chinese Medicine OR Traditional Medicine, Chinese OR Traditional Tongue Assessment OR Traditional Tongue Diagnosis OR Zhong Yi Xue OR TCM OR Drugs, Chinese Herbal OR Chinese Drugs, Plant OR Chinese Herbal Drugs OR Plant Extracts, Chinese OR Chinese Herbal OR Tang OR Fang OR Prescription) | 153788 |
| 3 | AB=(Randomized Controlled Trial OR RCT OR Randomly OR Randomized OR Controlled Clinical Trial OR Clinical Trial OR Clinical Study)AB=(Randomized Controlled Trial OR RCT OR Randomly OR Randomized OR Controlled Clinical Trial OR Clinical Trial OR Clinical Study) | 2199674 |
| 4 | #1AND #2 AND #3 | 22 |
| Sinomed database | | |
| 1 | "多囊卵巢综合征"[中文标题:智能] OR( "多囊卵巢综合症"[中文标题:智能] OR "多囊性卵巢综合症"[中文标题:智能] OR "多囊性卵巢综合征"[中文标题:智能]) | 15890 |
| 2 | "肥胖型"[中文标题:智能] OR "肥胖"[中文标题:智能] | 29984 |
| 3 | "中医"[中文标题:智能] OR( "中西医"[中文标题:智能] OR "中药"[中文标题:智能] OR "中草药"[中文标题:智能] OR "中成药"[中文标题:智能] OR "复方"[中文标题:智能] OR "法"[中文标题:智能] OR "自拟"[中文标题:智能] OR "氏"[中文标题:智能] OR "汤"[中文标题:智能] OR "方"[中文标题:智能] OR "散"[中文标题:智能] OR "丹"[中文标题:智能] OR "胶囊"[中文标题:智能] OR "丸"[中文标题:智能] OR "颗粒"[中文标题:智能] OR "剂"[中文标题:智能]) | 2364920 |
| 4 | ( "随机对照试验"[摘要:智能] OR "随机"[摘要:智能] OR "对照"[摘要:智能] OR "临床研究"[摘要:智能] OR "临床试验"[摘要:智能] OR "临床对照"[摘要:智能] | 3263909 |
| 5 | #1 AND #2 | 1010 |
| 6 | #3 AND #4 AND #5 | 204 |
| CNKI database | | |
| 1 | TI=(多囊卵巢综合征 + 多囊卵巢综合症 + 多囊性卵巢综合征 + 多囊性卵巢综合症) AND TI=(肥胖 + 肥胖型) AND AB=(随机对照试验 + 随机 + 对照 + 临床研究 + 临床试验 + 临床对照) AND TI=(中医 + 中西医 + 中药 + 中草药 + 中成药 + 复方 + 法 + 自拟 + 氏 + 汤 + 方 + 散 + 丹 + 胶囊 + 丸 + 颗粒 + 剂) | 323 |
| VIP database | | |
| 1 | M=(多囊卵巢综合征 OR 多囊卵巢综合症 OR 多囊性卵巢综合征 OR 多囊性卵巢综合症) AND R=(随机对照试验 OR 随机 OR 对照 OR 临床研究 OR 临床试验 OR 临床对照) AND T=(中医 OR 中西医 OR 中药 OR 中草药 OR 中成药 OR 复方 OR 法 OR 自拟 OR 氏 OR 汤 OR 方 OR 散 OR 丹 OR 胶囊 OR 丸 OR 颗粒 OR 剂) | 204 |
| Wanfang database | | |
| 1 | 题名:(多囊卵巢综合征 OR 多囊卵巢综合症 OR 多囊性卵巢综合征 OR 多囊性卵巢综合症) AND 题名:(肥胖 OR 肥胖型) AND 摘要:(随机对照试验 OR 随机 OR 对照 OR 临床研究 OR 临床试验 OR 临床对照) AND 题名:(中医 OR 中西医 OR 中药 OR 中草药 OR 中成药 OR 复方 OR 法 OR 自拟 OR 氏 OR 汤 OR 方 OR 散 OR 丹 OR 胶囊 OR 丸 OR 颗粒 OR 剂) | 312 |
